# Linking gastrointestinal microbiota and metabolome dynamics to clinical outcomes in paediatric haematopoietic stem cell transplantation

**DOI:** 10.1186/s40168-022-01270-7

**Published:** 2022-06-10

**Authors:** Gintare Vaitkute, Gordana Panic, Dagmar G. Alber, Intan Faizura-Yeop, Elaine Cloutman-Green, Jonathan Swann, Paul Veys, Joseph F. Standing, Nigel Klein, Mona Bajaj-Elliott

**Affiliations:** 1 Infection, Immunity and Inflammation Section, UCL Great Ormond Street Institute of Child Health, London, WC1N 1EH UK; 2 Department of Surgical Biotechnology, UCL Division of Surgery and Interventional Science, UCL, London, NW3 2PF UK; 3 Department of Metabolism, Digestion and Reproduction, Imperial College London, London, SW7 2AZ UK; 4 School of Human Development and Health, Faculty of Medicine, University of Southampton, Southampton, SO17 1BJ UK; 5 Sheffield Children’s NHS Foundation Trust, Sheffield, S10 2TJ UK; 6 Great Ormond Street Hospital NHS Foundation Trust, London, WC1N 3JH UK

**Keywords:** Gut microbiota, Haematopoietic stem cell transplantation, Paediatric, Microbiota dynamics

## Abstract

**Background:**

Haematopoietic stem cell transplantation is a curative procedure for a variety of conditions. Despite major advances, a plethora of adverse clinical outcomes can develop post-transplantation including graft-versus*-*host disease and infections, which remain the major causes of morbidity and mortality. There is increasing evidence that the gastrointestinal microbiota is associated with clinical outcomes post-haematopoietic stem cell transplantation. Herein, we investigated the longitudinal dynamics of the gut microbiota and metabolome and potential associations to clinical outcomes in paediatric haematopoietic stem cell transplantation at a single centre.

**Results:**

On admission (baseline), the majority of patients presented with a different gut microbial composition in comparison with healthy control children with a significantly lower alpha diversity. A further, marked decrease in alpha diversity was observed immediately post-transplantation and in most microbial diversity, and composition did not return to baseline status whilst hospitalised. Longitudinal trajectories identified continuous fluctuations in microbial composition, with the dominance of a single taxon in a significant proportion of patients. Using pam clustering, three clusters were observed in the dataset. Cluster 1 was common pre-transplantation, characterised by a higher abundance of *Clostridium XIVa*, *Bacteroides* and *Lachnospiraceae*; cluster 2 and cluster 3 were more common post-transplantation with a higher abundance of *Streptococcus* and *Staphylococcus* in the former whilst *Enterococcus*, *Enterobacteriaceae* and *Escherichia* predominated in the latter. Cluster 3 was also associated with a higher risk of viraemia. Likewise, further multivariate analysis reveals *Enterobacteriaceae*, viraemia, use of total parenteral nutrition and various antimicrobials contributing towards cluster 3, *Streptococcaceae*, *Staphylococcaceae*, *Neisseriaceae*, vancomycin and metronidazole contributing towards cluster 2. *Lachnospiraceae*, *Ruminococcaceae*, *Bifidobacteriaceae* and not being on total parenteral nutrition contributed to cluster 1. Untargeted metabolomic analyses revealed changes that paralleled fluctuations in microbiota composition; importantly, low faecal butyrate was associated with a higher risk of viraemia.

**Conclusions:**

These findings highlight the frequent shifts and dominations in the gut microbiota of paediatric patients undergoing haematopoietic stem cell transplantation. The study reveals associations between the faecal microbiota, metabolome and viraemia. To identify and explore the potential of microbial biomarkers that may predict the risk of complications post-HSCT, larger multi-centre studies investigating the longitudinal microbial profiling in paediatric haematopoietic stem cell transplantation are warranted.

Video abstract.

**Supplementary Information:**

The online version contains supplementary material available at 10.1186/s40168-022-01270-7.

## Introduction

Haematopoietic stem cell transplantation (HSCT) is a curative procedure for a variety of haematological, immunological and metabolic conditions. HSCT comprises a conditioning regimen, which includes chemotherapy with or without radiotherapy and/or antibodies prior to infusion followed by engraftment of donor or self-stem cells (allogeneic/autologous HSCT) [1]. Autologous HSCT in this cohort encompasses gene therapy and CAR T cell treatments, whereby the patient’s immune cells are modified ex vivo followed by a reinfusion of these cells. The procedure is, however, not without complications; infections (bacterial, viral and fungal) and graft-versus-host disease (GvHD), characterised by T cell-mediated tissue damage to target organs including skin, liver and the gastrointestinal (GI) tract, continue to be major causes of morbidity and mortality post-transplantation [2–4].

Multiple studies, predominantly in adult allogeneic HSCT, have profiled the gut microbiota linking microbial composition and function to clinical outcomes. Overall, depletion of obligate anaerobes, such as a loss of *Lachnospiraceae* and *Ruminococcaceae* families, and a decrease in diversity are commonly observed [5–8]. Mancini et al. observed low alpha diversity in adults 10 days post-transplantation, which correlated to an increased risk of GvHD within 30 days, whilst the presence of > 5% of *Enterobacteriaceae* at admission correlated with an increased risk of sepsis [9]. Most recently, Peled et al. also linked higher alpha diversity during the peri-engraftment period to a decreased risk of death in three adult HSCT cohorts, whereas Rolling et al. linked changes in the mycobiome, specifically the expansion of *Candida parapsilosis* complex species expansion, to a worse survival [10, 11].

Domination by a single taxon is a common feature in both adult and paediatric HSCT. Domination by *Enterococcus* or *Proteobacteria* has been linked to a greater risk of developing bacteraemia with vancomycin-resistant *Enterococcus* in adults [5, 6]. In paediatric acute lymphoblastic leukaemia, domination with *Enterococcaceae* or *Streptococcaceae* during chemotherapy preceded bloodstream infections (BSI) in the subsequent phase of chemotherapy [12]. BSI-causing strains dominated the gut and preceded the episodes by a median of 17 days [13].

HSCT patients are also prone to viral infections, which can lead to complications including viral pneumonitis and post-transplant proliferative disease. Cytomegalovirus (CMV) viraemia, in particular, is associated with an increased risk of overall mortality in the first year post-transplant [14, 15]. Haak et al. found a higher abundance of butyrate-producing bacteria at engraftment, which was associated with a lower likelihood of viral respiratory lower tract infections in adult HSCT [16]. Increasing evidence suggests that commensal microbiota/metabolites can modulate host inflammation and promote immune tolerance against a variety of viral pathogens [17]. Although molecular details are lacking, increasing evidence supports the hypothesis that gut bacterial composition and diversity prior to and during the HSCT procedure are key determinants of clinical outcomes post-transplantation.

Changes in microbial metabolites during HSCT parallel changes in microbial composition. Numerous studies have highlighted the crucial role of microbially derived short-chain fatty acids (SCFA) in modulating host glucose homeostasis, gut integrity and immune function [18]. Low levels of butyrate (4-carbon SCFA) pre-transplant increase the risk of BSI 30 days post-HSCT [19]. Additionally, Markey et al. found lower plasma concentrations of butyrate in patients who developed GvHD [20]. Interestingly, butyrate restoration mitigates murine GvHD, demonstrating a protective role of this specific SCFA in GvHD [21]. However, a cautionary note is required as Golob et al. recently highlighted a disparate role of butyrate; the authors identified a potential association between the presence of butyrogenic bacteria and the development of refractory GvHD [22].

Despite the advances in adults, studies investigating the gut microbial dynamics in paediatric HSCT are limited. Biagi et al. were the first to report the loss of alpha diversity and a decrease in *Faecalibacterium* and *Ruminococcus* genera in a paediatric cohort [23]. A decrease in total SCFAs was followed by recovery within the first 100 days post-transplantation [23]. Another study found that HSCT and associated treatment led to a progressive decrease in propionate (3-carbon SCFA) and butyrate in the 2 weeks following HSCT, and this was linked to greater anaerobic antibiotic exposure [24]. Similarly, patients with GvHD exhibited a loss of anti-inflammatory *Clostridia*, which was in turn associated with the use of anaerobic antimicrobials [25]. Additionally, the baseline microbiota of patients who go on to develop sinusoidal obstruction syndrome appears to be less diverse in comparison with patients free of this adverse outcome [26].

Recently, Ingham et al. identified associations between microbial families, immune biomarkers and clinical outcomes in 36 patients [27]. A high abundance of *Ruminococcaceae* was linked to a rapid reconstitution of natural killer and B cells, low risk of GvHD and mortality [27]. In contrast, higher abundances of facultative anaerobes, such as *Enterobacteriaceae*, were linked to an inflammatory environment [27].

At present, no data investigating the dynamic changes in the structure and function of the microbiota in a UK paediatric HSCT cohort is available. The aims of the current prospective single-centre study were to characterise the dynamics of GI microbiota and its corresponding metabolome and to link these findings to the outcomes including infections and GvHD post-transplantation. Collectively, our study offers additional insights into how diet and antimicrobial therapies contribute to perturbations in the microbiota post-treatment sequelae in a transplant setting.

## Methods

### Patient characteristics and sample collection

Sixty-four patients (median age, 5 years (0.4–14)) undergoing HSCT at Great Ormond Street Hospital, London, were recruited, and faecal samples were collected weekly during the inpatient stay (532 samples in total). Fifty-seven patients underwent allogeneic, whilst 7 patients underwent autologous HSCT (autologous encompassed gene therapy and CAR T cell treatments, whereby the patient’s immune cells were modified ex vivo followed by re-infusion). Preparative regimens were as follows: reduced-intensity conditioning included treosulfan/fludarabine or fludarabine/melphalan or reduced-intensity busulfan/fludarabine (targeted busulfan area under the curve of 45 to 65 mg × h/L). Myeloablative protocols were myeloablative busulfan/fludarabine (targeted busulfan area under the curve, > 70 mg × h/L) or treosulfan/fludarabine/thiotepa. GvHD grading (gut/skin) was done according to the Glucksberg criteria. Medication administrations, including antimicrobials, were also recorded. Prophylactic ciprofloxacin was administered from day − 8/− 10 until discharge or central venous line removal in younger patients. Viraemia was defined as a single positive detection of any virus (Epstein-Barr virus (EBV)/cytomegalovirus (CMV)/adenovirus/human herpesvirus 6 (HHV6)/parvovirus) via PCR of a blood sample, regardless of viral load. CMV, adenovirus and EBV were the most commonly detected in the cohort. Patients received both enteral and total parenteral nutrition (TPN). Further cohort characteristics are detailed in Additional file 1: Table S1.

Nine healthy controls were also studied. Healthy controls were not matched to the patients and consisted of stool samples from 5 females and 4 males with a median of 8 years (4–14), sampled once. Healthy controls did not receive antibiotics 6 months prior to sample collection. Upon receipt, all faecal samples were thoroughly mixed, aliquoted and frozen at − 80 °C until required.

### DNA extraction and 16S rRNA gene sequencing

Faecal samples were extracted using a Qiagen QIAamp DNA stool kit as per the manufacturer’s instructions with the following modifications. Once buffer ASL was added, the sample was heated to 95 °C. Homogenisation was performed with 8 bead-beating steps (Lysing Matrix E; MP Biomedicals) and 60-s rest on ice in between each step (60 s, 50 osc, TissueLyser LT). DNA was eluted in 200 μL of AE buffer and stored at − 20 °C until further processing. Primers spanning the V3–4 regions of the 16S ribosomal RNA gene including an Illumina adaptor, an 8-nucleotide barcode and a region-specific primer were utilised (341F (5#-CCTACGGGNGGCWGCAG-3#); 806R (5#-GGACTACHVGGGTWTCTAAT-3#)). Cycling conditions were 95 °C for 3 min followed by 30 cycles of 95 °C for 30 s, 54 °C for 30 s and 72 °C for 1 min followed by a final extension of 72 °C for 10 min. A mock community consisting of DNA from 8 bacterial species (D6305, Zymo, USA) and > 20 extraction controls were amplified and sequenced in parallel. PCR products were purified using Agencourt AMPure XP beads as per the manufacturer’s instructions (0.7×, Beckman Coulter). Amplicon quantity and quality were confirmed using the Agilent Tapestation (2200, Agilent) and the NEBNext Library Quant Kit for Illumina (NEB). Pooled libraries were spiked with 10% PhiX and sequenced using the V2 kit (500 cycles) on the Miseq sequencing platform (Illumina).

### 16S rRNA gene sequence processing and analysis

Data were demultiplexed, and sequence analysis was performed using mothur (V1.35.1) [28]. Unique reads were aligned using a region-specific Silva bacterial database (release 128) [29]. Chimera-free sequences (VSEARCH) were classified using the mothur-formatted Bayesian RDP database [30]. Mitochondria, archaea, chloroplast, eukaryota and unknown sequences were removed. Bacterial operational taxonomic units (OTUs) with a higher abundance in samples than in extraction controls were not considered contaminants, whereas those with a higher abundance in extraction controls than in samples were considered contaminants and were removed.

The dataset was subsampled without replacement to an equal depth of 2100 reads per sample in R for the comparisons of alpha diversity. Shannon effective entropy was calculated using Rhea in R by exponentiating Shannon entropy [31]. Further data analysis was conducted using vegan, phyloseq and microbiome packages in R (version 3.5.1). T-distributed stochastic neighbour embedding (t-SNE) plots were generated using the tsnemicrobiota package in R using ‘Bray’ distance. The term ‘stochastic’ is used to refer to seemingly random motion, such as the movements of patients between dominations.

### Clustering into community state types

Raw data were imported into the package Deseq2 and transformed using the function ‘varianceStabilizingTransformation’. Samples were assigned to community state types [CST(s)] by partitioning around medoid clustering using the function ‘pam’ in the package cluster based on a Jensen-Shannon distance. The number of clusters was determined by using the gap statistic evaluation and silhouette width quality validation (Additional file 2: Fig. S1). R code was adapted from Ingham et al. [27]. The results were then evaluated in an NMDS ordination (Additional file 2: Fig. S1). In individuals with two allogeneic transplants, only samples from the first transplant were used for producing CST assignment, transition and Cox models. In the individual with three transplantations, only samples relating to the allogeneic HSCT were utilised.

### Transition models

We further assessed the patients’ movements between CSTs during transplantation by using transition models. To investigate the dynamics of cluster progression over time, we subset the dataset to samples collected in the first 5 weeks, starting at day − 7 relative to transplantation, as the number of patients remaining in the hospital steadily decreased after week 5. In the case of more than one sample being collected within a week, the first sample was retained. A transition matrix containing frequencies of transitions between respective CSTs was generated and plotted using the qgraph package in R. The function ‘sojourn.msm’ was used to estimate the mean sojourn times for each CST. The R code used in this analysis was adapted from Stewart et al. [32].

### Time-dependent Cox models

To evaluate the associations between CSTs and clinical outcomes, right-censored time-dependent Cox models were used. Using GvHD (≥ grade II) or viraemia as the dependent variable, we performed a univariate analysis with the following independent variables: sex, age, diagnosis (malignant), more than one transplant, conditioning (myeloablative), cell source, serotherapy (in vivo), Shannon diversity and CST. Models for GvHD were also adjusted for graft manipulation (in vitro). Multivariate analysis with significant variables was then performed. A univariate model was also run using viraemia as a dependent variable and taxon dominance (> 30%) in CST3 as an independent variable (vs non-dominant samples in other CSTs). Covariates included in the models were selected a priori from domain knowledge before running the model. To reduce confounders, only samples from individuals receiving an allogeneic HSCT were used in time-dependent Cox models (478 samples; 57 patients). A *p*-value of ≤ 0.05 was considered significant. Repeated measures were adjusted using a robust sandwich estimator. Cox models were run using the survival package in R [33]. Azithromycin, clarithromycin and erythromycin were classed as macrolides; ciprofloxacin and moxifloxacin as quinolones; and ceftazidime, co-amoxiclav, imipenem, meropenem and piperacillin-tazobactam as broad-spectrum beta lactams. GvHD analysis only included samples from allogeneic HSCT patients, grades ≥ 2 were classed as clinically relevant GvHD and GvHD was analysed as a time-dependent outcome.

### Sparse partial least squares (sPLS) and canonical correspondence analysis (CCA)

We first assessed the correlations between numerical clinical variables by sPLS using the mixOmics package [34]. sPLS allowed us to integrate microbial data with the clinical data and perform multiple regressions. The number of components was chosen based on the scree plot for each principal component analysis (PCA), and the model was run in regression mode. Hierarchical clustering was then performed based on the sPLS model using Pearson correlation and complete linkage (Additional file 3: Fig. S2).

CCA was then performed using both continuous and categorical variables (clinical outcomes/baseline variables/antimicrobial administrations). In contrast to sPLS, CCA models relationships between OTUs and clinical variables bi-directionally. The data was chi-square transformed, followed by weighted linear regression on all variables of interest. The values are then used for canonical correspondence analysis by singular value decomposition. Only OTUs/variables with a correlation of > 0.3/< 0.3 were displayed in the CCA plot. Scripts for the analysis were adapted from Holmes and Ingham et al. [35, 36].

### Sample processing and ^1^H nuclear magnetic resonance (NMR) spectroscopy

Faecal samples were selected at random, and approximately 100 mg of sample was added to 700 μL of dH_2_0, homogenised using a bead beater (Precellys 24, Bertin Technologies, UK) with lysing matrix E beads, for 45 s at 6500 rpm twice and centrifuged at 10,000*g* for 20 min. The aqueous portion was removed and re-centrifuged under the same settings. An aliquot of the aqueous portion (630 μL) was mixed with 70 μL of pre-prepared phosphate buffer containing 1.5 M KH_2_PO_4_, 2 mM NaN_3_ and 1% trimethylsilylpropanoic acid in D_2_O. The mixture was vortexed and centrifuged at 10,000*g* for 10 min, and the resulting supernatant (600 μL) was added to a 5-mm NMR tube. Quality controls were included in each run, comprising a pool of each sample. A 600-MHz Bruker Avance III spectrometer was used to perform the spectroscopy. A standard one-dimensional NMR experiment was performed on each sample using the NOE pulse sequence and water suppression. The raw spectra were automatically baseline- and phase-corrected and calibrated to trimethylsilylpropanoic acid using Topspin 3.2 (Bruker Biospin). The spectra were then imported into MATLAB (version R2018b; MathWorks Inc., USA), and the redundant spectral regions (corresponding to water and trimethylsilylpropanoic acid) were removed. Manual alignment to the median spectrum and quality control spectra was performed using recursive segment-wise peak alignment. Finally, the spectra were normalised using a probabilistic quotient approach.

### Spectral data analysis

PCA was performed to summarise the overall variance in the dataset using whole spectra, whether metabolites were subsequently identified or not. Of the 439 samples, 14 outliers were identified and removed due to poor spectral quality. Orthogonal projection to latent structures (OPLS) and OPLS-discriminant analysis (OPLS-DA) methods were used to identify the biochemical variation related to continuous and categorical variables, respectively. Only models with positive *Q*^2^*Y* values, which are indicative of a model’s predictive ability, were further tested for significance using permutation testing (1000 permutations, *p*-value threshold: *p* ≤ 0.05). To identify which metabolites significantly contributed to the model, *p* ≤ 0.05 was used as a cut-off for the calculated *p*-values of each peak (correlation of spectral feature to the predictive component of the model). The corresponding metabolites were then identified using an in-house database and statistical total correlation spectroscopy using MATLAB. Several regions (potential drug residues) corresponding to 1.07–1.2 and 4.98–5.28 ppm were removed, and the baseline model was re-run; however, these regions did not influence the model (Additional file 4: Fig. S3). To investigate individual metabolites, peaks from the normalised spectra were integrated using trapezoidal numerical integration. Pearson correlation coefficient was used to correlate metabolite abundance and alpha diversity in baseline samples. Logistic regression was used to investigate the associations between viraemia and metabolites in allogeneic HSCT baseline samples. The model including metabolites and patient demographic and clinical characteristics was subjected to stepwise reduction using the Akaike criterion. Following this, the remaining metabolites were used as independent predictors alongside any significant baseline characteristics.

## Results

### Temporal changes in the alpha diversity of the gut microbiota during HSCT

**Fig. 1:**
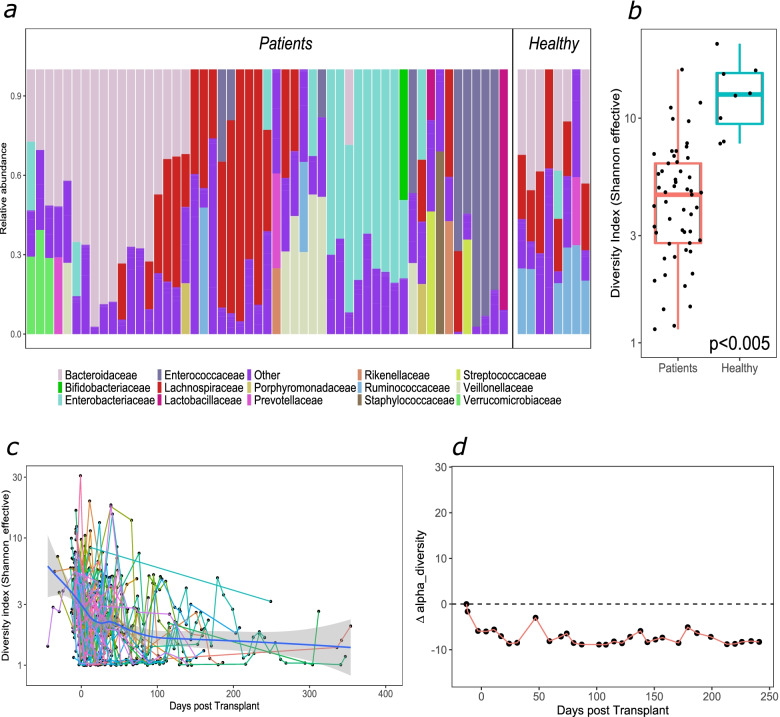
Baseline taxonomic composition and longitudinal changes in alpha-diversity in patients undergoing allogeneic HSCT. **a**) Relative abundance family level taxonomic plot (Only taxa with relative abundance of >10% are labelled) and **b**) alpha diversity of patient baseline samples (*n*=53) and unmatched healthy control samples (*n*=8). ***<0.001 Mann-Whitney test **c**) Alpha diversity throughout transplantation (shaded area- 95% CI) and **d**) delta changes in alpha diversity from baseline for an individual undergoing allogeneic HSCT (patient X)

We compared the microbial composition of allogeneic HSCT patients at baseline (first sample upon admission) to healthy controls (Fig. 1a). Patient samples were characterised by a distinct lack of obligate anaerobes, specifically *Lachnospiraceae* and *Ruminococcaceae*. Domination (≥ 30% relative abundance) with certain families, including *Enterobacteriaceae* and *Enterococcaceae*, was also a common feature—44/64 patients were dominated by either taxon at some point during post-transplantation. The microbial composition of autologous baseline samples exhibited similar characteristics to allogeneic baseline samples (Additional file 5: Fig. S4). Alpha diversity was significantly lower for the majority of patients at baseline in comparison with healthy controls, although some individual samples were comparable (Fig. 1b). Whilst most patients will have begun prophylactic antimicrobials, alpha diversity did not differ by baseline variables including diagnosis, sex and age (Additional file 6: Fig. S5). Following transplantation, a further decrease in alpha diversity was observed, most notably immediately post-transplantation (Fig. 1c). Diversity profiles of individual patients were stochastic (Fig. 1c); the majority (44/64) did not return to their initial baseline diversity during inpatient stay (average, 66 days) (representative patient X in Fig. 1d).

### Perturbations in the gut microbial composition and individual families during HSCT

**Fig. 2:**
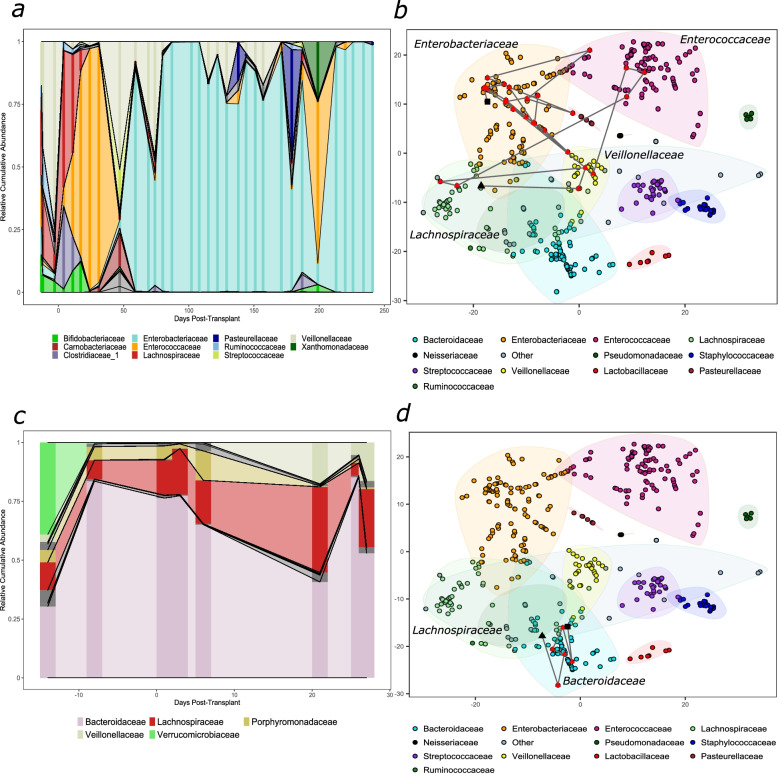
Longitudinal microbial taxonomies and trajectories during allogeneic HSCT. Taxonomic plots for two allogeneic HSCT patients **a**) X andc) Y throughout hospitalisation at a family level. Only families with an overall abundance of >10% are labelled. Darker vertical bars reflect sample collection points, abundances between these are inferred. (**b** & **d**) t-SNE plots of all samples collected in the study. Each sample is coloured by its predominant taxon. The lines represent a trajectory for patients shown in (**a**) and (**c**). A black triangle signifies the first collected sample and the black square- the last. ‘Other’ is composed of several infrequently dominant taxa

Next, we explored the longitudinal changes in gut microbial composition. A t-SNE plot was used to display the through-time variation in the microbial landscape. As seen with alpha diversity (patient X; Fig. 1d), compositional profiles were highly individualistic with domination a common phenomenon post-transplantation (patient X; Fig. 2a).

Most patients were observed to follow stochastic trajectories (illustrated by the compositional shifts in patient X; Fig. 2a, b). Interestingly, some patients (for example, patient Y; Fig. 2c, d) exhibited more stable trajectories and started to recover their initial composition before discharge. The majority (58/64) of the patients did not return to their initial microbiota composition. This is supported by a comparison of Bray-Curtis dissimilarities of the last collected samples for each patient with respect to their respective baseline sample, where most samples are more different (> 0.5) than similar (< 0.5) to their baseline sample (Additional file 7: Fig. S6).

Within the cohort, families that consistently dominated included *Enterococcaceae*, *Enterobacteriaceae*, *Staphylococcaceae* and *Streptococcaceae*. A gradual decrease in the abundance of *Bacteroidaceae* and *Lachnospiraceae* and a time-dependent increase in the overall abundance of *Enterococcaceae* and *Enterobacteriaceae* were observed (Additional file 8: Fig. S7). Overall, *Enterococcaceae* predominance increased approximately 2 weeks post-transplantation; in contrast, the relative abundances of *Enterobacteriaceae*, *Staphylococcaceae* and *Streptococcaceae* increased gradually, with *Enterobacteriaceae* abundance highest several months post-transplantation. As *Enterococcus* dominance has been previously linked to adverse clinical outcomes in adult populations, we next assessed if variables, including medications and underlying clinical features, were associated with *Enterococcus* dominance [5, 7]. Both myeloablative conditioning (HR 2.14, 95%CI 1.45–3.14; *p* = 0.03) and the use of macrolides (HR 2.04, 95%CI 1.33–3.14, *p* = 0.05) were associated with a higher risk for *Enterococcus* dominance; however, significance was lost in a multivariate Cox model (Additional file 9: Table S2).

### Community state type dynamics during HSCT

**Fig. 3:**
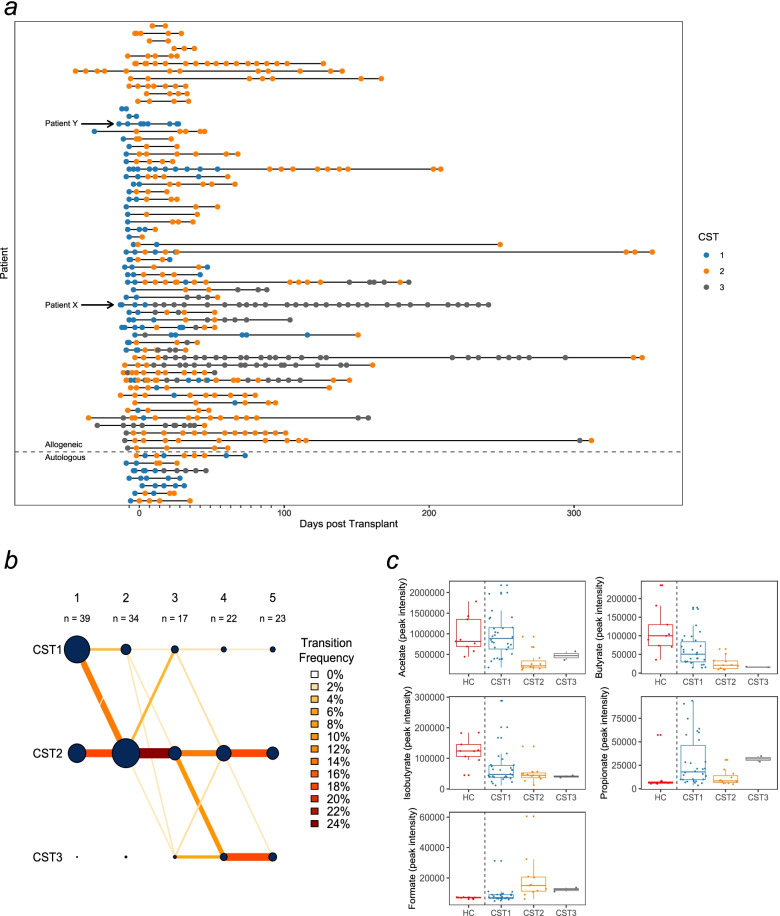
Community state type transitions and metabolome makeup at baseline. **a**) A timeline of all samples collected in the study, coloured by the CST they have been classified into. Extra demarcations on the x-axis denote weekly intervals. **b**) A transition model showing the progression of samples through each CST at each time point starting at day -7 relative to transplantation (week 1) to week 4 post-transplantation. Line colour intensity is indicative of transition frequencies, and the node sizes correspond to sample size within each node. **c**) SCFA and formate in baseline samples split by their respective CST

Patient samples were also partitioned into CST types. CST1 exhibited higher abundances of *Lachnospiraceae* and *Bacteroidaceae* and can be considered broadly ‘healthier’, as samples in this CST were more consistent with previously published healthy microbial profiles and healthy controls were classified as CST1 [37, 38]. CST2 showed high abundances of *Staphylococcaceae* and *Streptococcaceae* whilst CST3 showed high abundances of *Enterobacteriaceae* and *Enterococcaceae*. It is important to emphasise that taxa were not exclusive to any one cluster (Additional file 10: Fig. S8). CST1 was predominant prior to transplantation (51% of CST1 samples), whilst CST2 and CST3 were seen less frequently pre-transplantation (14% and 6% of CST2 and CST3, respectively) and mostly observed post-transplantation (Fig. 3a). A proportion of patients (23%) remained in the same CST throughout the procedure, whilst some (17%) transitioned between all CSTs. The majority of baseline CST1 patients eventually transitioned to either CST2 or CST3 during their inpatient stay.

We also investigated the frequency of transitions between CSTs in the first month post-transplantation (Fig. 3b). The frequency of CST1 decreased, with the majority of samples switching from CST1 to CST2 in the first week post-transplantation, whilst most transitions to CST3 occurred 2 weeks post-transplantation. Transitions from CST2/3 to a healthier CST1 were rare, and no transitions were recorded 3 weeks post-transplantation. Overall, the self-transition probabilities of CST2 and CST3 were high (0.72; 0.82), whereas CST1 was less stable (0.4). Interestingly, CST3 was associated with a higher risk of viraemia (HR 2.07 (CI 0.29–2.52); *p* = 0.01; Additional file 11: Table S3), that is, when samples were observed to be in cluster 3, they were twofold more likely to develop viraemia than in cluster 1. A Kaplan-Meier plot details the risks in CSTs (Additional file 12: Fig. S9). Although *Enterobacteriaceae* dominance showed a propensity for a higher risk of viraemia, this trend did not reach statistical significance in the present study (Additional file 13: Table S4). No CSTs or other variables were associated with GvHD (Additional file 14: Table S5). The average time a sample remained in each CST was variable, with CST1 being 8 days, CST2 26 days, and CST3 37 days.

To gain potential insights into the functionality of each CST, we measured the abundance of SCFA and formate in each stool sample at baseline using NMR (Fig. 3c). Baseline samples belonging to CST1 contained comparable amounts of acetate (2C SCFA) and formate to healthy controls (also in CST1), yet lower amounts of butyrate and isobutyrate and higher propionate. In contrast, CST2 and CST3 stool samples contained lower amounts of acetate, butyrate and isobutyrate compared to healthy controls/CST1 and higher formate.

### Faecal metabolome profiling during HSCT

**Fig. 4:**
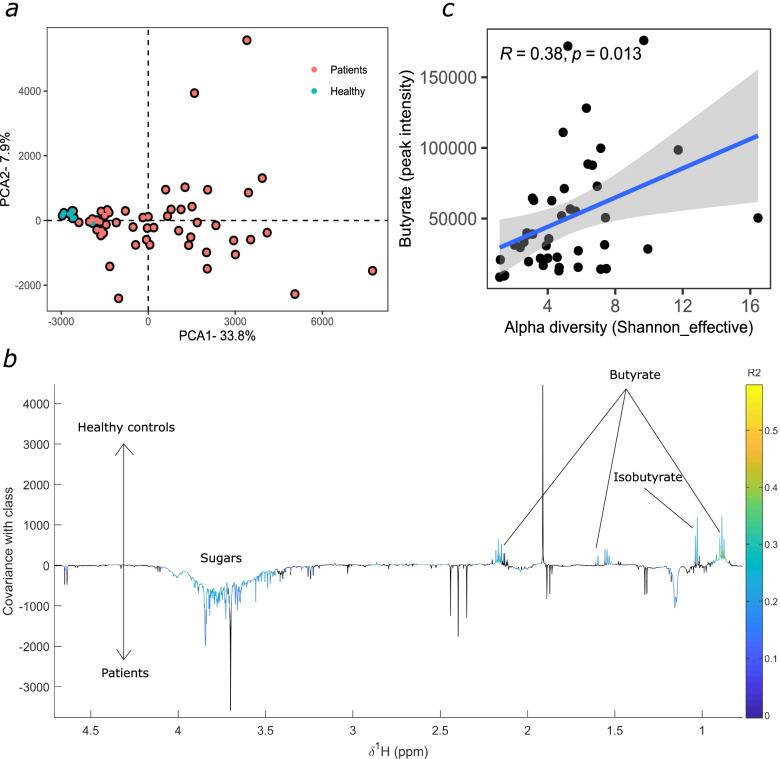
Metabolomic profiling of allogeneic HSCT patients at baseline versus Healthy controls. **a**) PCA between unmatched healthy controls (*n*=9) and patient baseline samples (*n*=53). **b**) An OPLS-DA coefficients plots of the model comparing healthy controls to baseline HSCT samples. Significant peaks are coloured. **c**) Butyrate correlates to alpha diversity at baseline (*n*=43) (*R*=0.38; *p*=0.01)

A PCA comparing untargeted ^1^H NMR stool profiles of the baseline patient and healthy control samples was performed. The analysis highlighted the heterogeneity in patient samples and the relatively low variance in the healthy control samples (Fig. 4a).

OPLS-DA analysis was performed to identify metabolic variation between the sample classes (*N* = 62; 1 predictive and 3 orthogonal components; *Q*^2^*Y* = 0.03; *p* < 0.01), from which an OPLS coefficient plot was produced to identify significantly contributing metabolites. The separation was largely driven by short-chain and branched-chain fatty acids and sugars (Fig. 4b). Faecal butyrate and isobutyrate were lower in the patients compared to the healthy controls whilst glucose was higher in the patient stools. Several peaks, which may be a result of drug metabolism, were removed to confirm that the model remains significant (*N* = 62; 1 predictive and 3 orthogonal components; *Q*^2^*Y* = 0.03; *p* < 0.003; Additional file 4: Fig. S3).

We next correlated the baseline SCFA to alpha diversity and found a positive correlation to butyrate (Fig. 4c). Additionally, butyrate at baseline was associated with a lower risk of viraemia (OR 0.99, standard error 1.49E−05; probability 0.49; *p* = 0.02; Additional file 15: Table S6), that is, for every unit increase in butyrate, the risk of subsequent viraemia decreases by a factor of 0.99.

Metabolite PCA analyses of samples in the first month post-transplantation indicated that overall, the week before transplantation is dissimilar to weeks post-transplantation (Additional file 16: Fig. S10). Longitudinally, acetate and butyrate continued to decrease, and glucose increased over the first month post-transplantation; trends were less clear for other metabolites (Additional file 17: Fig. S11).

### Multivariate associations of diet and antimicrobial usage with gut microbial profiling and clinical parameters

**Fig. 5:**
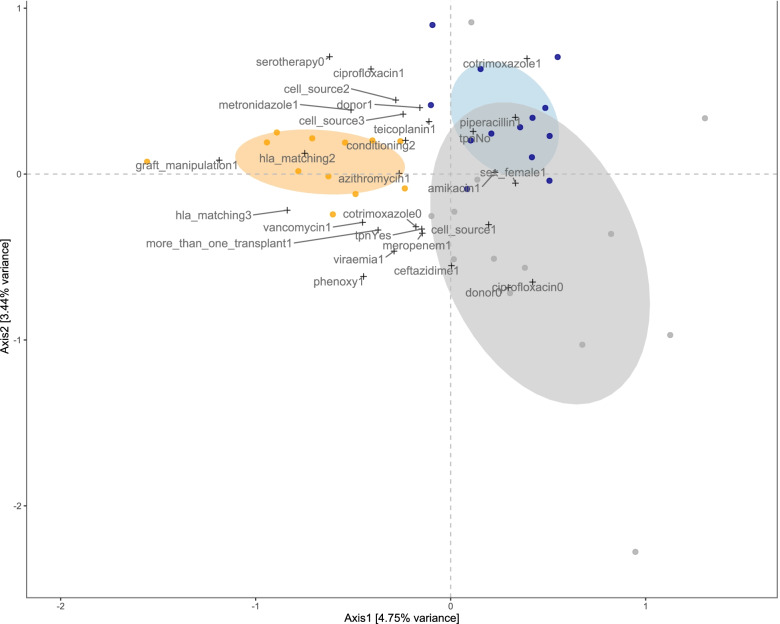
Multivariate associations of the gut microbiota with clinical parameters in allogeneic HSCT. Canonical correspondence analysis (CCA) relating gut microbial abundances (circles) to categorical (+) clinical parameters (Axis 1 vs 2). The plot displays variables and bacterial families with a score > 0.3/< − 0.3 on at least one of the first three CCA axes, displayed on axis 1 versus 2. The ellipses (60% confidence interval) correspond to the clusters identified by the sPLS-based hierarchical clustering (cluster 1: blue; cluster 2: orange; cluster 3: grey). Abbreviations: cell source2- PBMC; cell source 3- cord; donor1-unrelated matched;  conditioning2- reduced intensity; hla-matching2- mismatched; hla-matching3- haploidentical; phenoxy- phenoxymethylpenicillin; donor0- related matched; cell source1- bone marrow

Next, two multivariate approaches were employed to gain insight into how the gastroinstestinal microbiota abundances co-vary with clinical parameters, including TPN and antimicrobial administration. sPLS identified three clusters, loosely reflecting CSTs identified by pam (Additional file 3: Fig. S2). Amongst other taxa *Lachnospiraceae*, *Ruminococcaceae* and *Bifidobacteriaceae* as well as albumin contributed to cluster 1 and *Streptococcaceae*, *Staphylococccaceae* and *Neiserriaceae* contributed to cluster 2, whilst *Enterobacteriaceae*, *Lactobacillales* and age contributed to cluster 3 (Additional file 3: Fig. S2). We then used CCA, which allowed the inclusion of categorical baseline, clinical and outcome variables (Fig. 5).

Cluster 1 in CCA associated with the use of cotrimoxazole and piperacillin and a lack of TPN. Cluster 2 associated with the use of numerous antimicrobials including azithromycin, metronidazole and vancomycin. Additionally, cluster 2 included samples from patients with mismatched grafts (hla_matching2; hla_matching3) and those receiving in vitro graft manipulation (graft_manipulation1). Cluster 3 associated with the use of meropenem and ceftazidime, but not ciprofloxacin. It included samples from patients from matched donors (donor0) and those receiving a PBMC graft (cell_source1) as well as patients on TPN and those with viraemia. Consequently, we compared metabolites in patients on and off TPN at baseline and found a significant decrease (*p* = 0.05) in acetate levels in patients on TPN (Additional file 18: Fig. S12). A trend for a decrease in butyrate and glucose whilst a trend for increase in propionate and formate was also noted.

## Discussion

Our current understanding of the role and contribution of the gut microbiota on adverse outcomes in paediatric patients post-HSCT remains in its infancy. Herein, we report the first study from the UK investigating the microbiota-metabolome axis in 64 patients undergoing HSCT at Great Ormond Street Hospital, a major tertiary centre in London.

Our findings indicate that patients generally exhibit lower alpha diversity at baseline (pre-transplant) compared to healthy controls; most patients will have begun prophylactic antimicrobials at this time point; therefore, one may hypothesise that this is not a surprising finding. A further decrease in alpha diversity post-transplantation was common, and in most cases, microbial diversity did not return to pre-transplant baseline levels during the hospitalisation period, a feature consistent with reports in adult HSCT [5]. The absence of obligate anaerobes including *Ruminococcaceae* and *Lachnospiraceae* and the observed domination by a single taxon post-transplantation is likely due to extensive and prolonged exposure to antimicrobials, particularly the high anaerobe coverage received by the current cohort. Piperacillin-tazobactam and meropenem, which were frequently administered in our cohort, have been shown to impact obligate anaerobes in an adult HSCT cohort [39]. Taken together, the data suggests that antibiotic stewardship is a key determinant and therefore amenable for intervention for better microbial functional status and clinical outcome post-transplantation.

Consistent with previous reports in both adults and children, we identified the expansion of facultative anaerobes, including *Enterococcaceae*, *Enterobacteriaceae* and *Streptococcaceae* post-transplantation; adding support to the notion that despite the differences in transplant procedure and drug regimens between various hospital centres, expansion of facultative anaerobes is a salient feature post-HSCT transplantation [27, 36, 39, 40]. This is overall in line with another paediatric study; interestingly, the authors also identified the presence of a high *Lactobacillaceae* cluster [27], which was not seen in our cohort. The drivers for domination of specific families remain unknown; however, the post-transplant gut environment, due to conditioning regimens, diet and drug administration amongst other factors, is likely a major determinant. Although certain dominations are specific to the current cohort, the dynamics of the microbiota appear similar to that seen in adults. Indeed, several murine studies indicate that germ-free mice have defects in haematopoiesis and antibiotic-treated mice show multilineage repression of haematopoiesis [41, 42]. Staffas et al. recently demonstrated that intestinal microbiota contributes to haematopoiesis post-HSCT via improved dietary energy uptake in mice [43]. Studies to better understand the impact of specific taxon dominance on immune reconstitution in adult and paediatric post-HSCT are clearly warranted.

Plotting the samples in a t-SNE space illuminated a dynamic HSCT microbial landscape. Most individuals showed frequent stochastic movements, with multiple dominations throughout their inpatient stay and no return to their initial state (patient X; Fig. 2a, b). In contrast, a few patients exhibited relatively few transitions, and some returned close to their initial microbiota composition (patient Y; Fig. 2c, d). Similar observations about the lack of obligate anaerobes in the baseline samples and stochastic movements across the microbial landscape were also seen in the autologous patients. It is important to note that patient X received a broad-spectrum antibiotic prior to switching to CST3 and that the apparent instability of their microbiome may be in part due to longer hospitalisation and hence overall health post-transplant. At present, it is unclear why certain individuals return to their initial profile, whereas others do not, which warrants further investigation. Such behaviour may be an intrinsic feature, for example, a more diverse microbiota at the beginning of the treatment may be less susceptible to further modulation. The age of the patient, and therefore the maturation of the gut microbiota at the time of transplant, may also play a role in its resilience to continuous perturbation. Likewise, the observed stability or lack thereof may in part reflect the overall health of the patient post-transplant. Additionally, given that patients were followed up for variable amounts of time, this does not exclude the possibility that patients could have recovered at later time points.

Assuming that there is a single equilibrium value with a functionally optimal microbiota for each individual, an insult, such as a course of antibiotics, may lead to perturbation and thus a shift away from this optimum. It is tempting to hypothesise that with the number of continuing insults during HSCT, this single equilibrium value is gradually shifted away to a new value, and eventually, the landscape itself is altered [44, 45]. Palleja et al. showed that healthy adults are resilient to a short course of broad-spectrum antibiotics, as they return to near-baseline composition within 1.5 months [46]. Despite this, certain taxa remained undetectable 6 months post-treatment. Significant antimicrobial and immunosuppressant usage during HSCT have been found to have a profound impact on the microbial landscape [44, 45]. Given the high anaerobe cover in this cohort, it is not surprising that most patients in this study exhibited a perturbed microbial composition on admission and during treatment and did not return to their initial pre-transplant microbiota status during the observation period. Investigations into the optimal use of antimicrobials with a view of preserving the GI microbiota in this population are essential, and a study is already underway in an adult cohort (NCT03078010).

To identify the distinct bacterial community patterns within the population, the data were partitioned into three CSTs, each with a varied microbial composition. Transition models revealed time-dependent patterns such as transitions between CST2 and CST3 to CST1 becoming less common with time, with no such transitions observed after week 3. This suggests that there may be a critical time period after transplantation, during which the microbiota is able to return towards a healthier state. The microbiota around this period may also be more amenable to intervention. Both CST2 and CST3 appear to have high self-transition probabilities, making them more stable than CST1. This is similar to another study in adults, which suggests that the resilience of a biodiverse state is low during the post-HSCT period, with an observed self-transition probability of 49%, in comparison with our observation of 40% [5].

Importantly, we found that CST3 was associated with a higher risk of viraemia after transplantation. CST3 is a state with a complex composition, and on further analysis, we were unable to identify specific taxa responsible for this association, although *Enterobacteriaceae* was close to significance. A small sample size might be at play, or a higher risk of viraemia could be a result of a cumulative effect of several taxa. The composition of CST3 is complex and includes several *Proteobacteria* genera including *Klebsiella*, *Escherichia* and *Enterococcus*. It is unclear how *Enterococcus* and/or other taxa may contribute towards an increased risk of viraemia; however, this could be through indirect action via delayed immune re-constitution. Given the associations between the HSCT gut microbiota and immune cell dynamics, particularly, between white blood cells and anaerobes such as *Ruminococcus*, *Fecalibacterium* and *Akkermansia*, it is plausible that HSCT gut microbiota may impact immune reconstitution and, in turn, viraemia [47]. Additionally, the taxa may act indirectly through reactivation and/or delayed/impaired response to viral infection/reactivation. The commensal microbiota (through MAMPs engaging pattern recognition receptors) as well as derived metabolites are known to impact and generate optimal innate and adaptive immune responses important for controlling systemic viral infections as well as having an impact on viral-specific CD8 T cell memory in a murine model infected with CMV [48, 49]. Additionally, CST3 could simply be a marker of ‘poor’ gut health such as damage to the colonic mucosa, which may have an impact on viraemia development. Several recent publications link CMV reactivation to antimicrobial use in adult HSCT populations. Zhang et al. found an increased risk of CMV reactivation with the use of vancomycin, whereas Camargo et al. found the use of anaerobic antimicrobials in the first 2 weeks post-transplantation associated with a twofold risk of clinically significant CMV reactivation [50, 51]. Given the high use of vancomycin in this cohort, the impact of the microbiota and/or antimicrobials on viral reactivation and clinically significant viraemia would be of interest in the future.

Variations in microbial metabolites were also recorded during HSCT. This included reduced amounts of SCFA and BCFA butyrate and isobutyrate in the baseline patient samples versus healthy controls. Butyrate at baseline positively correlated to microbial diversity and was associated with a reduced risk of viraemia. Although the link to diversity has been previously observed, the association to the risk of viraemia is a novel finding [7, 24, 52, 53]. Butyrate exerts multi-fold effects on intestinal epithelial cell integrity and immune cell homeostasis; thus, higher levels may, to an extent, be indicative of a ‘healthier’ microbiota. This cohort may benefit from an intervention that aims to restore levels of SCFA either directly or through prebiotics, probiotics or postbiotics. Despite this, the administration of butyrate in in vitro models inhibits colonic stem cells from forming an intact epithelial monolayer; therefore, investigations into the most appropriate approaches for this cohort are warranted [54]. In contrast, glucose was significantly higher in the patient group. This may be an indication of a degree of gut damage/malabsorption and/or the result of the expansion of certain facultative anaerobes, specifically *Enterococcus* [55]. A recent study highlights that lactose drives *Enterococcus* expansion in a murine model; thus, an increase in glucose may be the result of lactose metabolism; however, either of these hypotheses require further investigation [56].

sPLS and CCA revealed potential associations between the three clusters and patient clinical parameters. Of interest, cluster 1, which was high in obligate anaerobes, was associated with the non-use of TPN and the use of cotrimoxazole and piperacillin. Detailed effects of TPN on GI microbiota have not been elucidated, although the use of TPN and higher GvHD incidence and worse survival have been reported [53]. There is an ongoing debate on whether enteral nutrition may be preferable over TPN for preserving the GI barrier integrity and ecology. The association with cotrimoxazole and piperacillin is less clear given that piperacillin is a broad-spectrum antimicrobial and has been shown to have an effect on obligate anaerobes [39]. Cotrimoxazole is a PCP prophylactic, and previous work has shown that it has a somewhat limited effect on the microbiota composition [57].

Cluster 2 associated with the use of multiple antimicrobials, including metronidazole, vancomycin and azithromycin, all known to be detrimental to the commensal gut anaerobes, likely contributing to the milieu of taxa observed in this particular cluster [46]. Finally, cluster 3 also associated with the use of various antimicrobials including meropenem and ceftazidime. Meropenem and piperacillin have a detrimental impact on obligate anaerobes whereas ceftazidime was found to have a moderate effect in a HSCT cohort [39]. Quinolones (e.g., ciprofloxacin) in contrast exert a marginal effect [39]. Despite this, given that no use of ciprofloxacin was associated with cluster 3, there is likely an effect in this cohort. Interestingly, in agreement with the CST findings, viraemia and TPN both contribute to cluster 3. Additionally, acetate was significantly lower in baseline samples of allogeneic HSCT patients receiving TPN than in those without TPN. Whilst the associations appear rational, further insight into the exact dynamics of antimicrobial administration and the use of TPN in this patient cohort requires a detailed investigation. Furthermore, given the associations between the type of donor and cluster 3, it would be of interest to also profile donor gut microbiota where feasible. The lack of associations between GvHD and the GI microbiota is somewhat surprising given the previous findings; however, it may be explained, to some extent, by the heterogeneity of the cohort, making a signal more difficult to identify.

In summary, the current study investigated longitudinal microbiota and metabolome in paediatric HSCT patients at a single centre. Despite differing conditioning and treatment regimens between transplant centres, several salient features between adult and paediatric HSCT were identified including domination by a single taxon and no return to baseline within the hospitalisation period. Our study is the first to suggest an association between microbiota and butyrate levels at baseline and risk of viraemia. Although our report is the largest longitudinal study in paediatric HSCT to date, given the heterogeneity of the patients, the sample size is relatively small, which prevents us from undertaking more in-depth analysis including the stratification of the patients by their underlying condition or age. We collected weekly samples during the hospital stay; therefore, the microbiota data is limited, and it is plausible that we were unable to fully capture all dominations, which could improve CST resolution. In order to comprehensively profile the microbiota, we sampled patients weekly; however, due to patient heterogeneity, such as discharge times, this led to irregular overall sampling times. It would be of interest to extend to similar sampling times for all patients in the future.

The impact of certain co-variates known to affect the microbiota such as nutrition and antimicrobial use underwent limited investigation; therefore, an in-depth study on their effects is necessary. Patients in our cohort frequently had co-infections; we were therefore unable to delineate the specific associations between specific viraemia, i.e., CMV/EBV and the microbiota. As a result of previous findings, associations between bacteraemia and the microbiota were also of interest in this cohort; however, given the low number of bacteraemias in this cohort, we were unable to interrogate this further.

The samples size of the healthy controls was relatively small in the present study; in addition, the HC were not followed longitudinally. We observed minimal variability in the clustering of the HC metabolomics profiles (unlike the patient samples), which highlights the marginal variability of these samples in comparison with patient samples; it is likely that a larger sample size of HC would be unlikely to provide a greater deal of additional information. Likewise, given that microbiota remains fairly stable once mature, repeat sampling was unlikely to add additional insights to the study. Finally, whilst 16S rRNA sequencing remains a useful way to taxonomically profile the microbiota, in order to gain a deeper understanding of microbial dynamics, future studies must employ metagenomic and metatranscriptomic approaches to gain molecular insights into microbial-host interactions. Despite these limitations, the study provides a reference point for further vital work in this population.

## Conclusions

Together, these findings are indicative of a disruption in the gut microbiota and a dysregulation of the host-microbiota metabolism. The intestinal microbiota is in a complex relationship with the mucosal epithelium whereby epithelial cells and the microbiota establish a state of equilibrium, which in turn facilitates optimal nutrient absorption as well as resistance to infections [58]. Chemotherapy-induced tissue damage, dietary input, the dampening of the immune system and the administration of antibiotics and other medications together are likely to disrupt this equilibrium, leading to the expansion of facultative anaerobes and the dysregulation in metabolites we observe [58]. Identification of potential mechanisms involved in butyrate/microbiota-mediated impact on viral pathogenesis/reactivation offers a novel axis to explore host-microbe communication. Further investigations into the dynamics of the gut microbiota during paediatric HSCT may inform us as to the best approach for microbiota-based therapeutics for this cohort.

## Supplementary Information

**Additional file 1: Table S1.** Cohort characteristics. ^1^Two patients had 2 transplants, one patient had 3 transplants. The first patient had a peripheral blood and a cord transplant, another had a bone marrow and a peripheral blood transplant. A single patient had 2 CAR T-cell infusions and an HSCT. ^2^Matched refers to a full HLA match (10/10; 12/12); ^3^Mismatched refers to a lesser HLA match. Haplo refers to a half HLA match to the patient. ^4^Transplant-related mortality is defined as mortality due to a complication other than a relapse following an HSCT. Antimicrobial administration refers to an antimicrobial given at any point during inpatient stay for HSCT.

**Additional file 2: Figure S1.** The number of clusters determined by using the gap statistic evaluation and silhouette width quality validation.

**Additional file 3: Figure S2.** sPLS regression of taxa and clinical parameters. *a)* Correlation circle plot for the first two sPLS dimensions (correlations > 0.3/< − 0.3 are shown). Grey circles indicate correlation radii at 0.5 and 1.0. Bacterial families are displayed as circles and are coloured according to the cluster they are affiliated with (cluster 1: blue; cluster 2: orange; cluster 3: grey). Variables situated perpendicularly to each other are not correlated. *b)* Loading plots of families with their contributions to component 1 and 2. The bars are coloured according to the cluster they affiliate with.

**Additional file 4: Figure S3.** Metabolomic profiling of HSCT patients at baseline *versus* Healthy controls. *a)* An OPLS-DA coefficients plots of the model comparing healthy controls to baseline HSCT samples with the peaks between 1.07-1.2 and 4.98-5.28 ppm removed. Significant peaks are coloured.

**Additional file 5: Figure S4.** Baseline taxonomic composition and alpha diversity in patients undergoing autologous HSCT. *a)* Relative abundance family level taxonomic plot and *b)* alpha diversity of patient baseline samples (*n* = 6) and unmatched healthy control samples (*n* = 8). Only taxa with relative abundance of >10% are labelled ***<0.001 Mann-Whitney test.

**Additional file 6: Figure S5.** Baseline alpha diversity of allogeneic samples (*n* = 53) stratified by diagnosis, age and sex. Mann-Whitney test. ns- non-significant.

**Additional file 7: Figure S6.** Bray-Curtis dissimilarity of the last collected sample for each patient in respect to the baseline sample.

**Additional file 8: Figure S7.** Fluctuating taxa landscape in the first 100 days post-transplantation. Relative abundance of taxa found to be dominant in the cohort during the first 100 days. The fitted line shows a local polynomial regression fit calculated using glm, with the grey region indicating the 95% CI.

**Additional file 9: Table S2.** Univariate and multivariate Cox models with *Enterococcus* domination (>30%) as the dependent variable. The 95% CI and *P* values were estimated using the robust sandwich estimator. *P* value of <0.05 was considered significant. Abbreviations: CI, confidence interval; HR, hazard ratio; -, not significant.

**Additional file 10: Figure S8.** Taxonomic composition of the CSTs *a)* Taxonomic CST composition of all samples (*n* = 540) *b)* Distribution of the top 7 taxa among the CSTs.

**Additional file 11: Table S3.** Univariate and multivariate Cox models with Viraemia as the dependent variable. The 95% CI and *P* values were estimated using the robust sandwich estimator. *P* value of <0.05 was considered significant.

**Additional file 12: Figure S9.** Kaplan-Meier plot of probability of viraemia in the first 100 days stratified by the microbiome CSTs.

**Additional file 13: Table S4.** Univariate Cox model with Viraemia as the dependent variable and dominance as the independent variable. The 95% CI and *P* values were estimated using the robust sandwich estimator. *P* value of <0.05 was considered significant. Dominance is classified as >30% Relative abundance.

**Additional file 14: Table S5.** Univariate Cox model with GvHD as the dependent variable. The 95% CI and *P* values were estimated using the robust sandwich estimator. *P* value of <0.05 was considered significant.

**Additional file 15: Table S6.** A logistic regression model with viraemia as the dependent variable. *P* value of <0.05 was considered significant.

**Additional file 16: Figure S10.** Metabolite PCA plot. Patient samples pre-transplant and over the first four weeks post-transplant (*n* = 114; range 19-26). Week -1 denotes days -7 to -1 relative to transplantation. Larger dots denote the centroid for each week.

**Additional file 17: Figure S11.** Metabolite profiles during the first five weeks of transplantation. Week -1 denotes days -7 to -1 relative to transplantation (sample range 19-26/week). The blue line indicates the mean for each week.

**Additional file 18: Figure S12.** Metabolites at baseline in samples from allogeneic HSCT patients on (*n* = 33) and off (*n* = 10) total parenteral nutrition. Kruskal-Wallis univariate comparison.

## Data Availability

Raw 16S rRNA files were deposited in the SRA depository (accession number: PRJNA818501; https://www.ncbi.nlm.nih.gov/sra/PRJNA818501). Raw NMR files were deposited in the MetaboLights depository (accession number: MTBLS4065; www.ebi.ac.uk/metabolights/MTBLS4065) [59]. To preserve the anonymity of the study participants, a full metadata file is not supplied. A complete metadata file can be obtained by contacting the lead authors subject to a data sharing agreement.

## Abbreviations

HSCT: Haematopoietic stem cell transplantation
GvHD: Graft-versus-host disease
SCFA: Short-chain fatty acids
GI: Gastrointestinal
BSI: Bloodstream infection
OTU: Operational taxonomic units
CST: Community state type
NMDS: Non-metric multidimensional scaling
OPLS: Orthogonal partial least-square
OPLS-DA: Orthogonal partial least-square discriminant analysis
PCA: Principal component analysis
sPLS: Sparse partial least squares
CCA: Canonical correspondence analysis
