# Supplementary material for: Linking gastrointestinal microbiota and metabolome dynamics to clinical outcomes in paediatric haematopoietic stem cell transplantation
Source: Microbiome. 2022 Jun 10;10:89. doi: 10.1186/s40168-022-01270-7 (PMC9185888; doi:10.1186/s40168-022-01270-7)
Supplement: Supplementary file 2 — Additional file 1: Table S1. Cohort characteristics. 1Two patients had 2 transplants, one patient had 3 transplants. The first patient had a peripheral blood and a cord transplant, another had a bone marrow and a peripheral blood transplant. A single patient had 2 CAR T-cell infusions and an HSCT. 2Matched refers to a full HLA match (10/10; 12/12); 3Mismatched refers to a lesser HLA match. Haplo refers to a half HLA match to the patient. 4Transplant-related mortality is defined as mortality due to a complication other than a relapse following an HSCT. Antimicrobial administration refers to an antimicrobial given at any point during inpatient stay for HSCT. [file 40168_2022_1270_MOESM2_ESM.docx]

**Table S1** Cohort characteristics

| Patient Characteristics (N =64) | No(%) |
| --- | --- |
| Age at transplant, median (range) | 5.3(0.4-14) |
| Age at transplantation, years |  |
| <2 | 16(25) |
| >2 | 48(75) |
| Sex |  |
| Male | 40(62) |
| Female | 24(38) |
| Underlying diagnosis |  |
| Haematological malignancy | 33(51) |
| Haematological non-malignancy/Other | 9(14) |
| Primary Immunodeficiency | 19(30) |
| Metabolic disease | 3(5) |
| Conditioning regimen |  |
| Myeloablative | 38(59) |
| Reduced intensity | 26(41) |
| Treatment^1^ |  |
| Allogeneic HSCT | 60(88) |
| 2x Allogeneic HSCT | 2(3) |
| Autologous HSCT | 8(12) |
| 2x Autologous HSCT + 1x Allogeneic HSCT | 1(2) |
| Stem cell source (Allogeneic HSCT) |  |
| Bone Marrow | 33(58) |
| Cord blood | 6(11) |
| Peripheral blood | 18(31) |
| Donor type |  |
| Matched Sibling/Family^2^ | 9(16) |
| Matched unrelated^2^ | 24(42) |
| Mismatched/Haploidentical^3^ | 24(42) |
| More than one transplant over lifetime |  |
| Yes | 9(14) |
| No | 55(86) |
| Mortality |  |
| Transplant-related^4^ | 11(17) |
| Relapse | 4(6) |
| Acute GvHD |  |
| Grade 0- I | 34(61) |
| Grade II -III | 21(37) |
| Patients with infection outcomes |  |
| Bacteraemia | 6(9) |
| Viraemia | 42(66) |
| Antimicrobial administration |  |
| Ciprofloxacin | 56(88) |
| Vancomycin | 46(72) |
| Piperacillin/tazobactam | 53(83) |
| Meropenem | 41(64) |
| Metronidazole | 11(17) |
| Co-amoxiclav | 12(19) |

^1^Two patients had 2 transplants, one patient had 3 transplants. The first patient had a peripheral blood and a cord transplant, another had a bone marrow and a peripheral blood transplant. A single patient had 2 CAR T-cell infusions and an HSCT.^2^Matched refers to a full HLA match (10/10; 12/12); ^3^Mismatched refers to a lesser HLA match. Haplo refers to a half HLA match to the patient. ^4^Transplant-related mortality is defined as mortality due to a complication other than a relapse following an HSCT. Antimicrobial administration refers to an antimicrobial given at any point during inpatient stay for HSCT.
