# Supplementary figures and images for: Linking gastrointestinal microbiota and metabolome dynamics to clinical outcomes in paediatric haematopoietic stem cell transplantation
Source: Microbiome. 2022 Jun 10;10:89. doi: 10.1186/s40168-022-01270-7 (PMC9185888; doi:10.1186/s40168-022-01270-7)

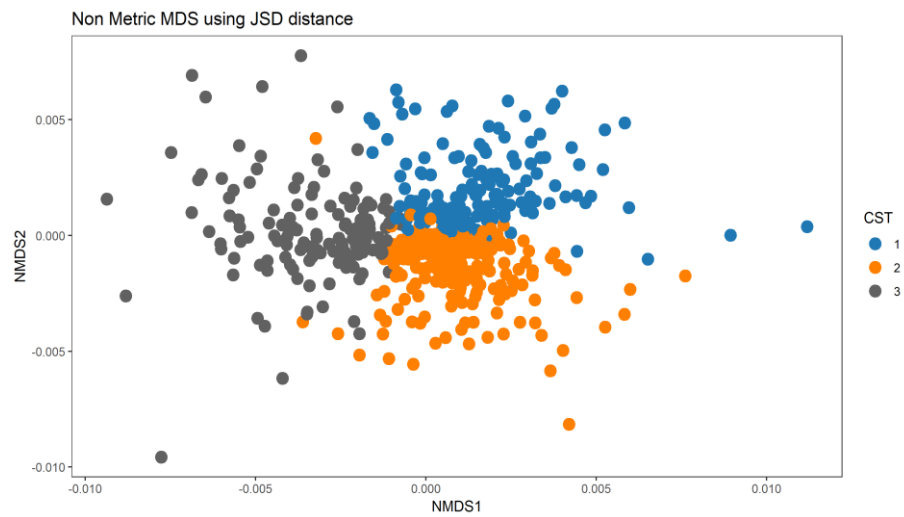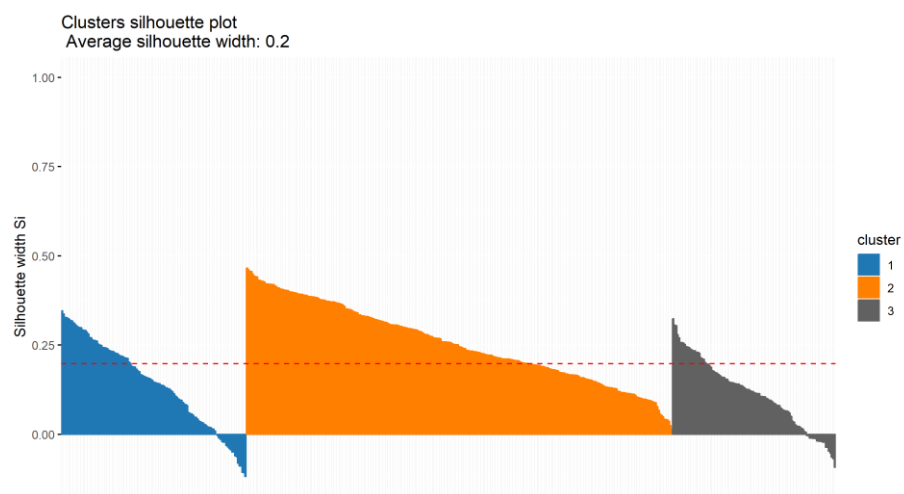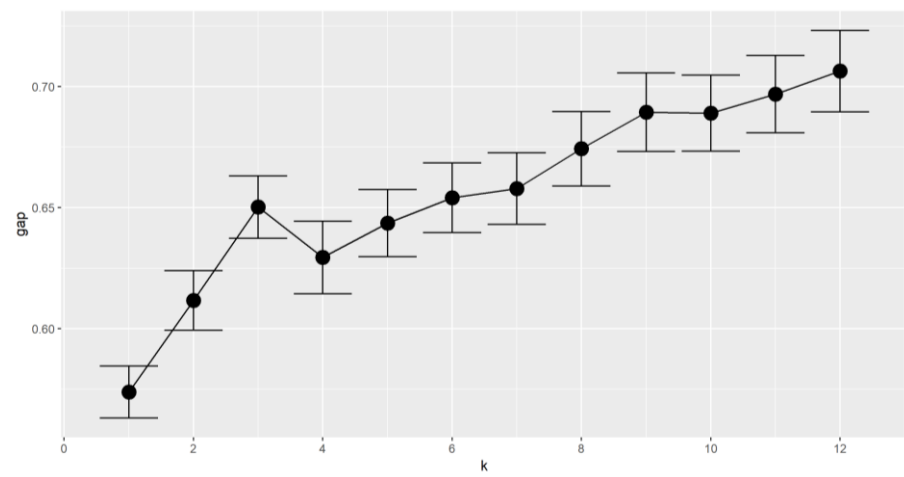

Supplement: Supplementary file 3 — Additional file 2: Figure S1. The number of clusters determined by using the gap statistic evaluation and silhouette width quality validation. [file 40168_2022_1270_MOESM3_ESM.pdf]

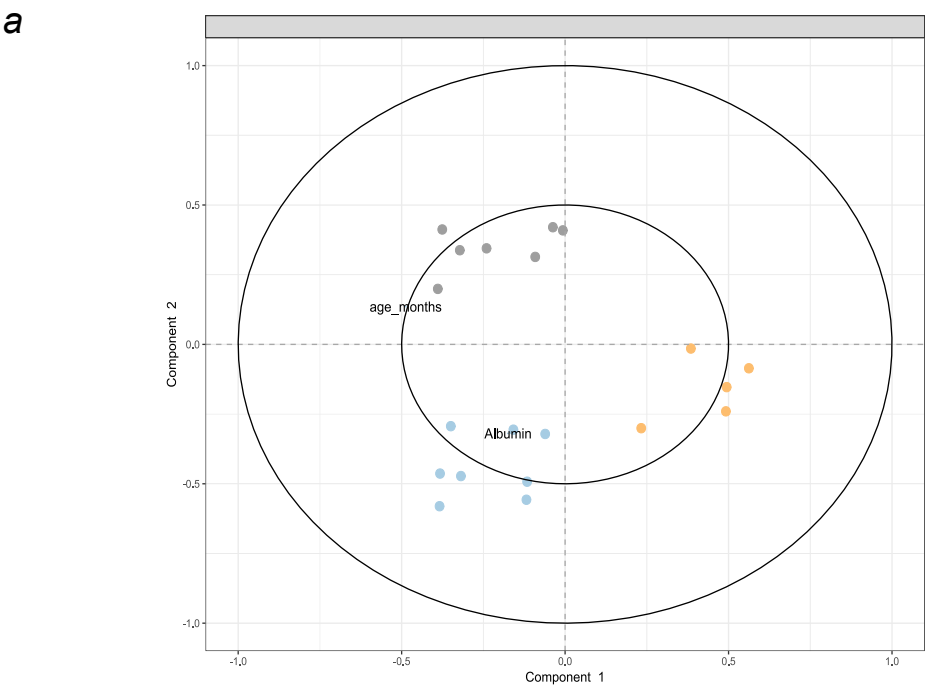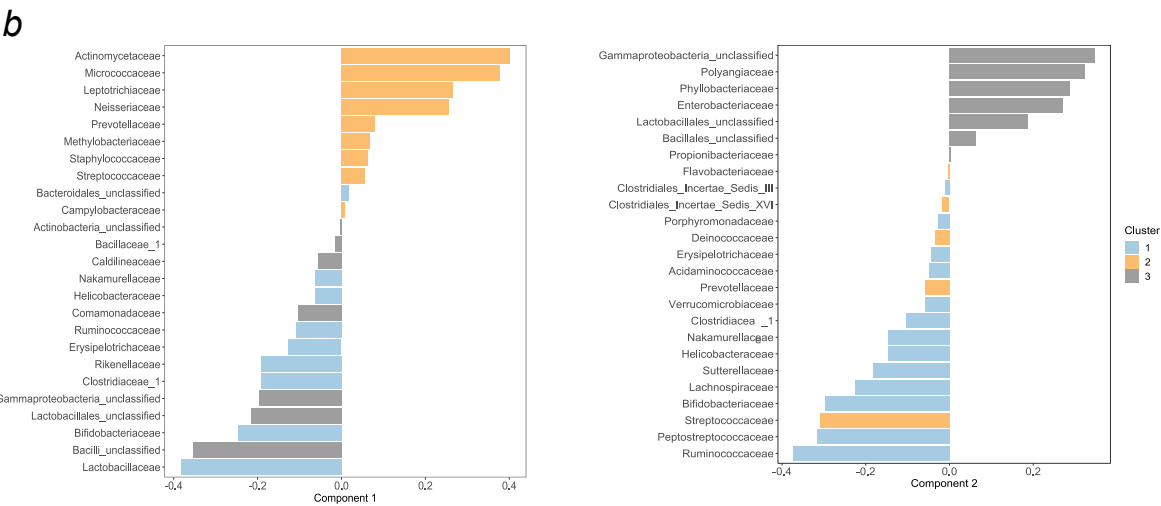

Supplement: Supplementary file 4 — Additional file 3: Figure S2. sPLS regression of taxa and clinical parameters. a) Correlation circle plot for the first two sPLS dimensions (correlations > 0.3/< − 0.3 are shown). Grey circles indicate correlation radii at 0.5 and 1.0. Bacterial families are displayed as circles and are coloured according to the cluster they are affiliated with (cluster 1: blue; cluster 2: orange; cluster 3: grey). Variables situated perpendicularly to each other are not correlated. b) Loading plots of families with their contributions to component 1 and 2. The bars are coloured according to the cluster they affiliate with. [file 40168_2022_1270_MOESM4_ESM.pdf]

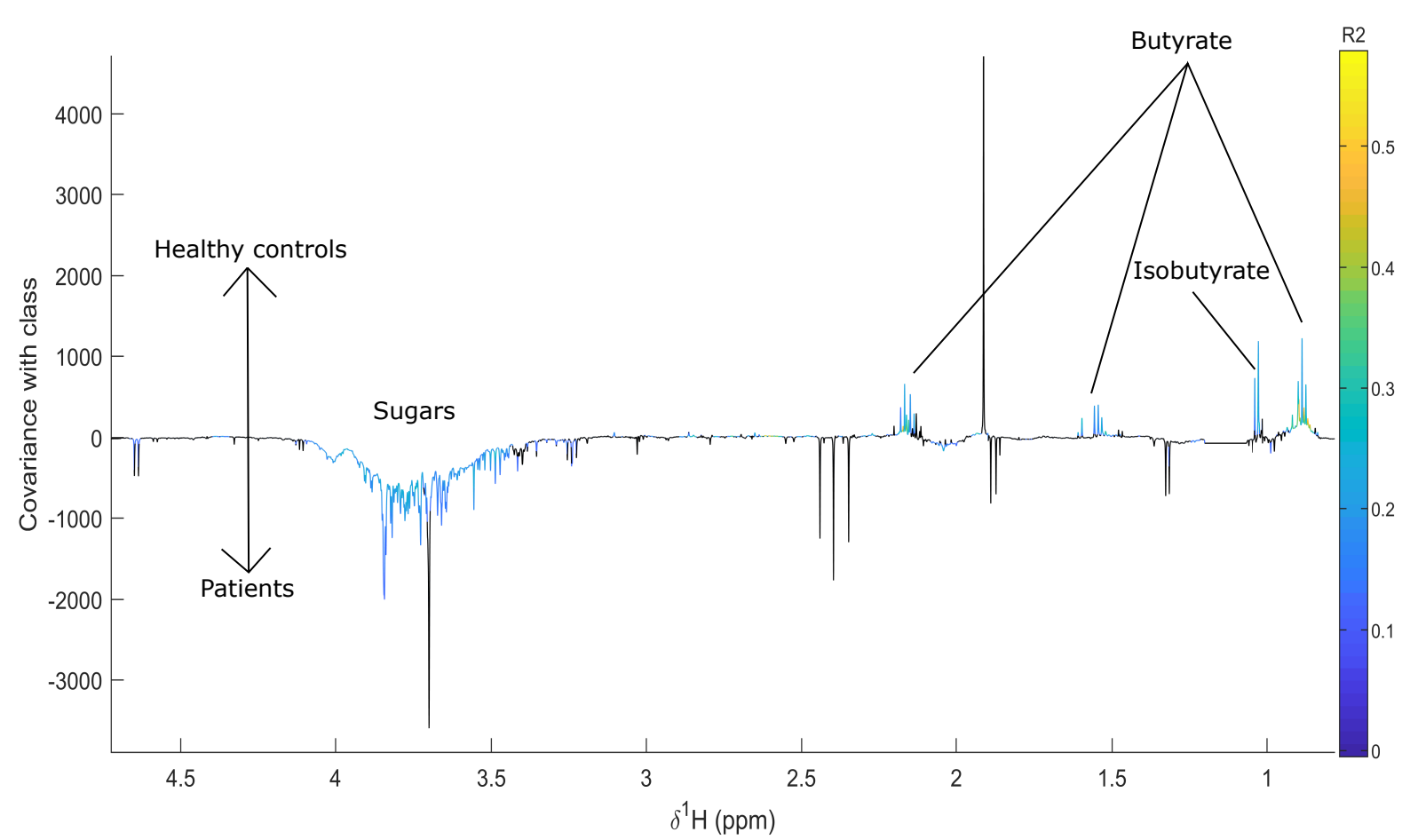

Supplement: Supplementary file 5 — Additional file 4: Figure S3. Metabolomic profiling of HSCT patients at baseline versus Healthy controls. a) An OPLS-DA coefficients plots of the model comparing healthy controls to baseline HSCT samples with the peaks between 1.07-1.2 and 4.98-5.28 ppm removed. Significant peaks are coloured. [file 40168_2022_1270_MOESM5_ESM.pdf]

**a**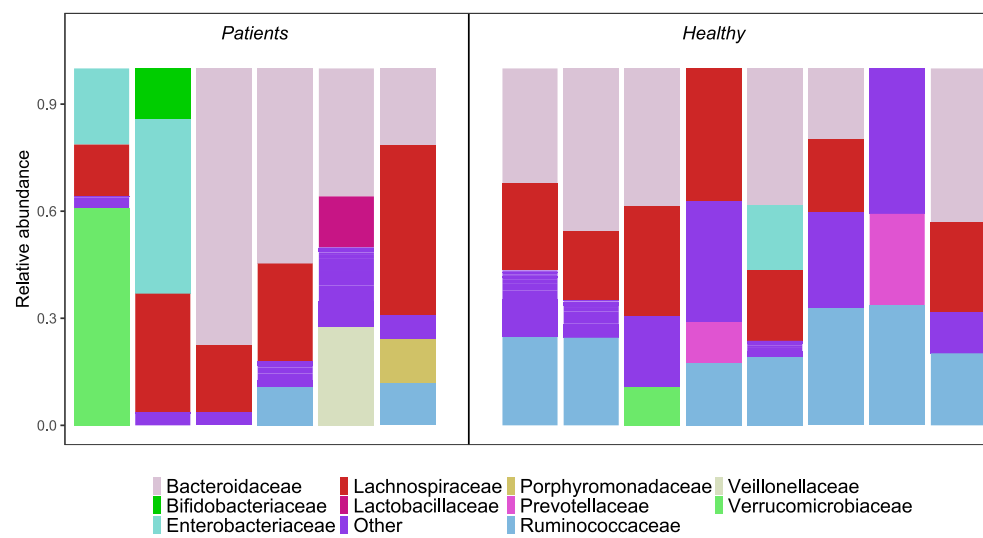**b**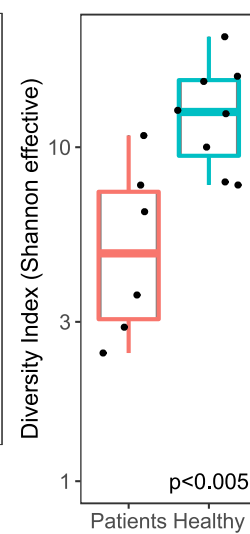

Supplement: Supplementary file 6 — Additional file 5: Figure S4. Baseline taxonomic composition and alpha diversity in patients undergoing autologous HSCT. a) Relative abundance family level taxonomic plot and b) alpha diversity of patient baseline samples (n = 6) and unmatched healthy control samples (n = 8). Only taxa with relative abundance of >10% are labelled ***<0.001 Mann-Whitney test. [file 40168_2022_1270_MOESM6_ESM.pdf]

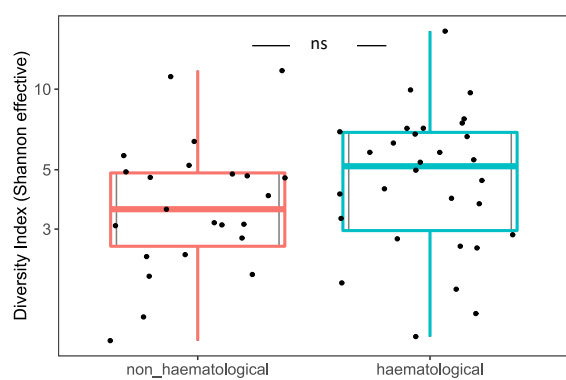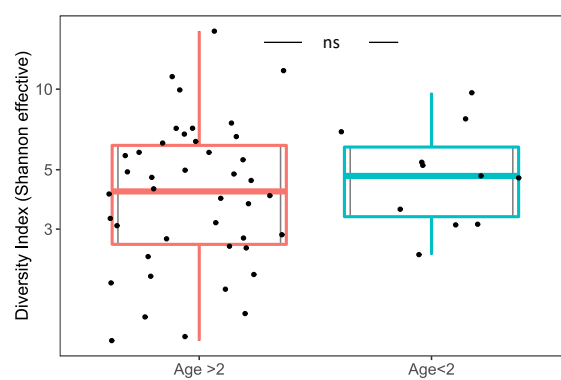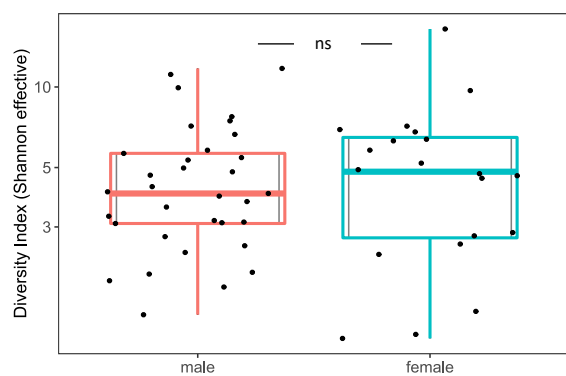

Supplement: Supplementary file 7 — Additional file 6: Figure S5. Baseline alpha diversity of allogeneic samples (n = 53) stratified by diagnosis, age and sex. Mann-Whitney test. ns- non-significant. [file 40168_2022_1270_MOESM7_ESM.pdf]

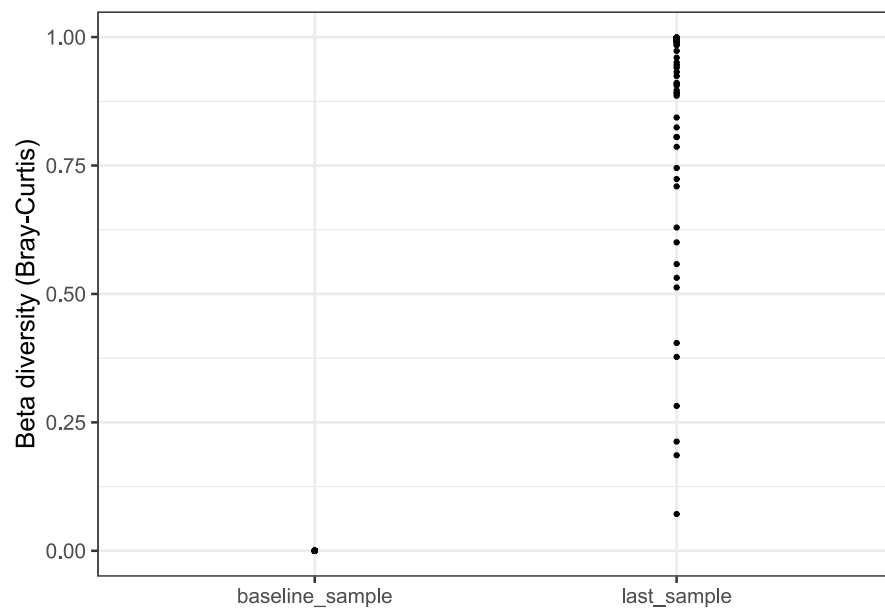

Supplement: Supplementary file 8 — Additional file 7: Figure S6. Bray-Curtis dissimilarity of the last collected sample for each patient in respect to the baseline sample. [file 40168_2022_1270_MOESM8_ESM.pdf]

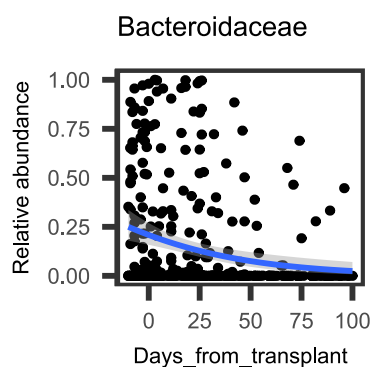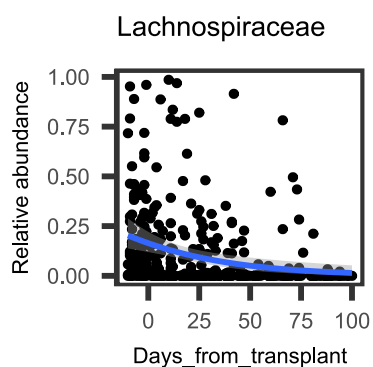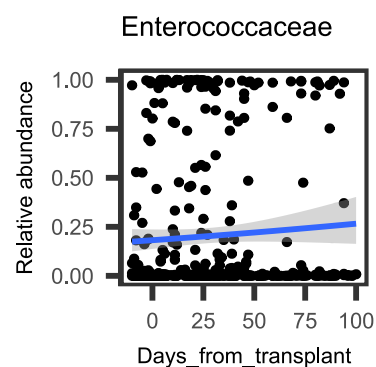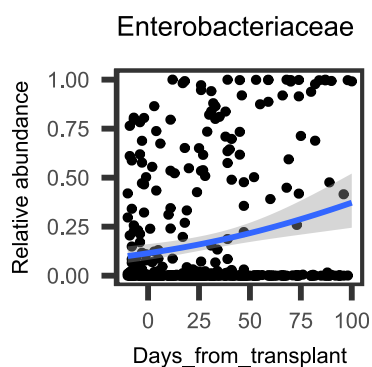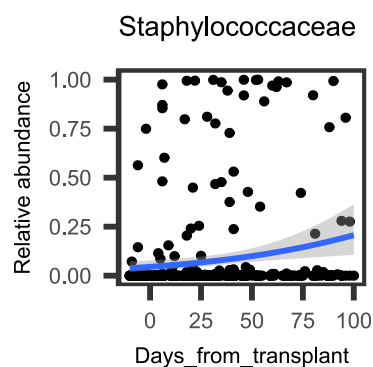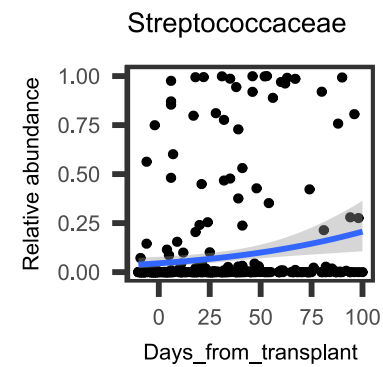

Supplement: Supplementary file 9 — Additional file 8: Figure S7. Fluctuating taxa landscape in the first 100 days post-transplantation. Relative abundance of taxa found to be dominant in the cohort during the first 100 days. The fitted line shows a local polynomial regression fit calculated using glm, with the grey region indicating the 95% CI. [file 40168_2022_1270_MOESM9_ESM.zip › 1-FigureS7.pdf]

**a**

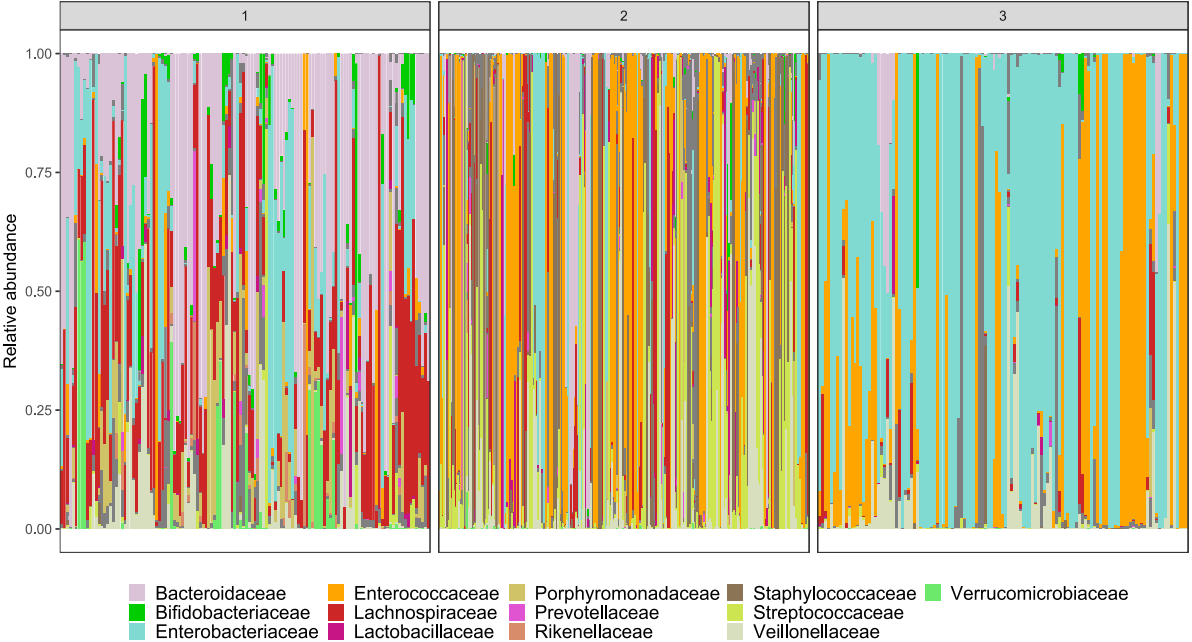

**b**

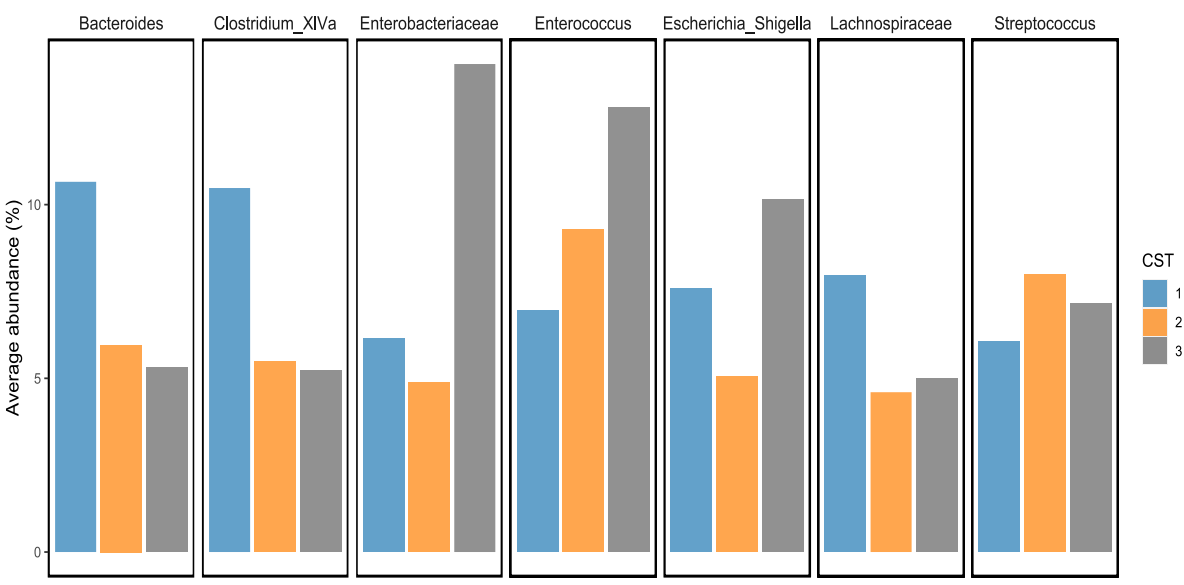

Supplement: Supplementary file 11 — Additional file 10: Figure S8. Taxonomic composition of the CSTs a) Taxonomic CST composition of all samples (n = 540) b) Distribution of the top 7 taxa among the CSTs. [file 40168_2022_1270_MOESM11_ESM.pdf]

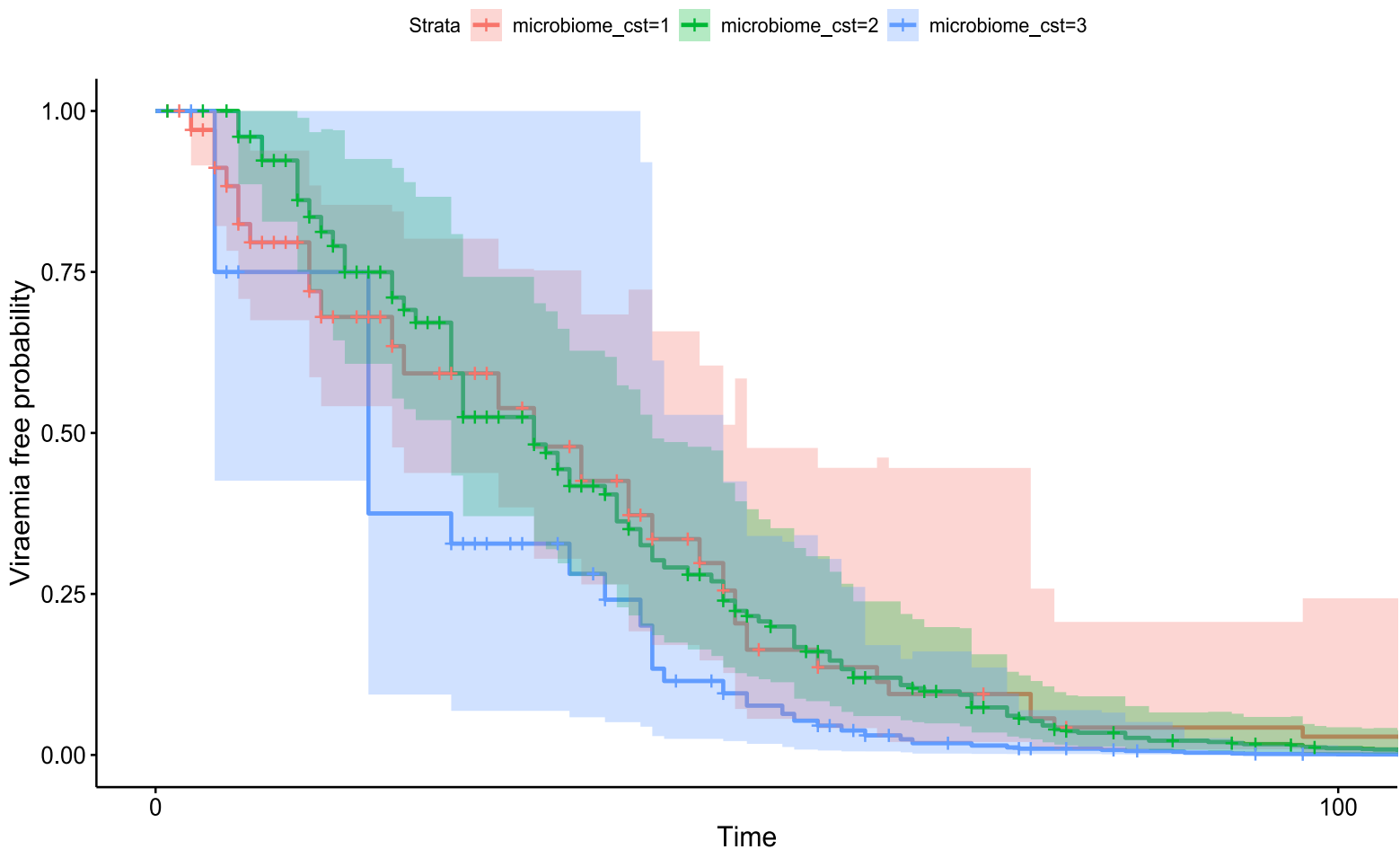

Supplement: Supplementary file 13 — Additional file 12: Figure S9. Kaplan-Meier plot of probability of viraemia in the first 100 days stratified by the microbiome CSTs. [file 40168_2022_1270_MOESM13_ESM.pdf]

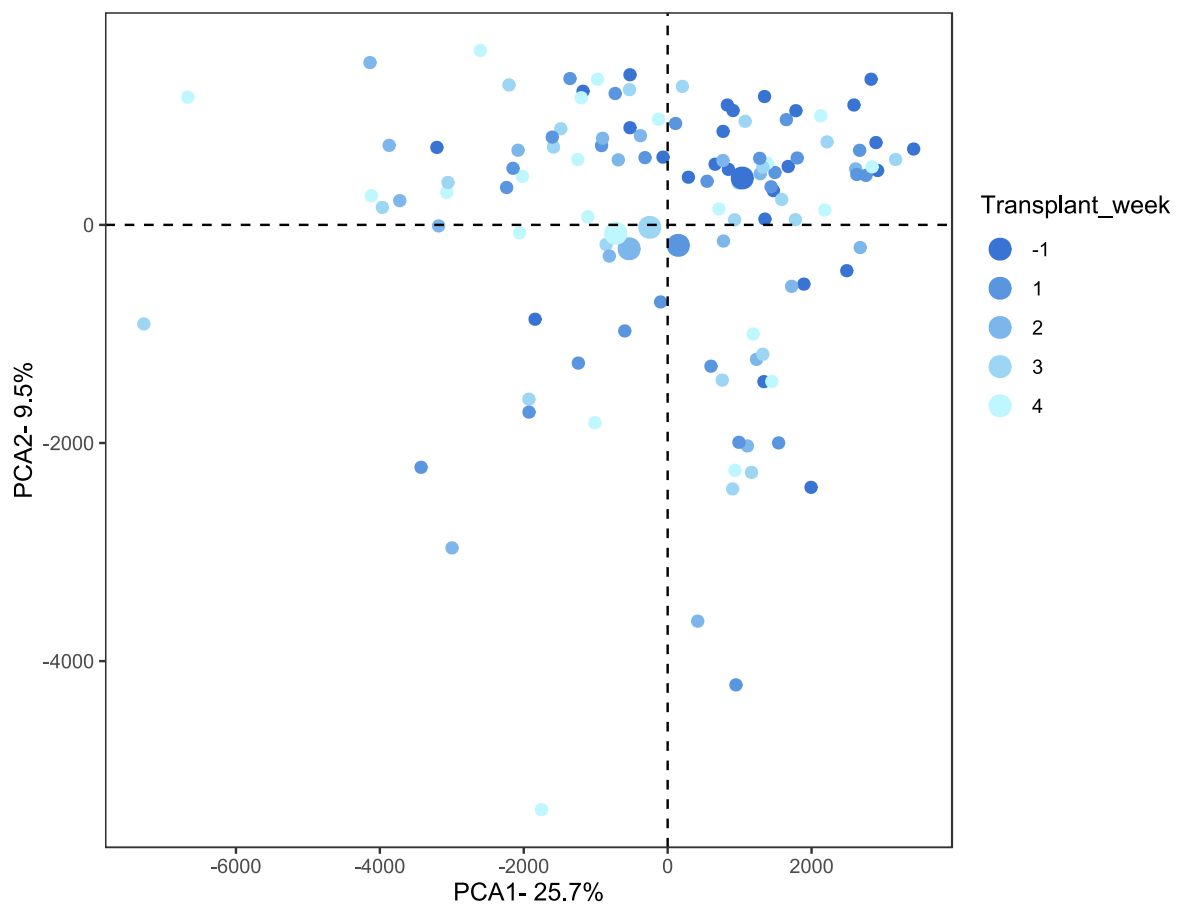

Supplement: Supplementary file 17 — Additional file 16: Figure S10. Metabolite PCA plot. Patient samples pre-transplant and over the first four weeks post-transplant (n = 114; range 19-26). Week -1 denotes days -7 to -1 relative to transplantation. Larger dots denote the centroid for each week. [file 40168_2022_1270_MOESM17_ESM.pdf]

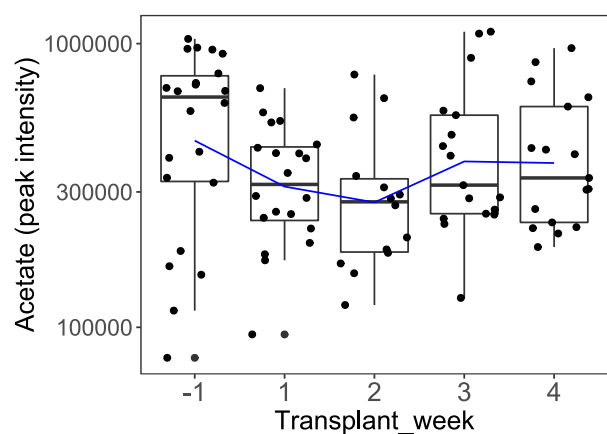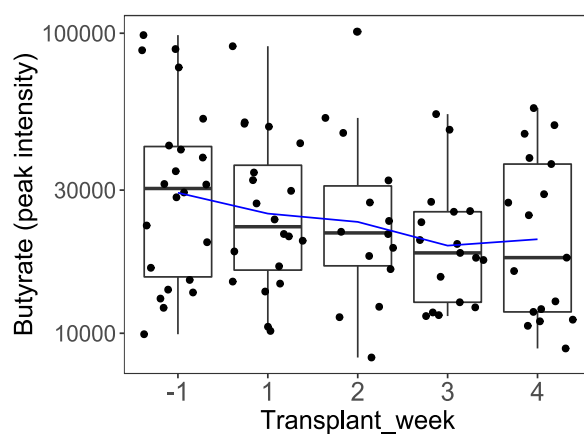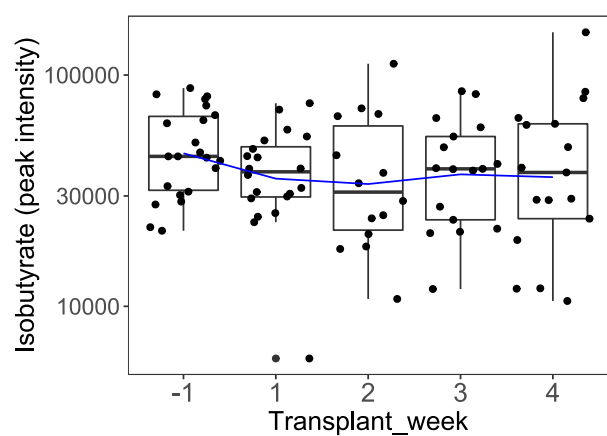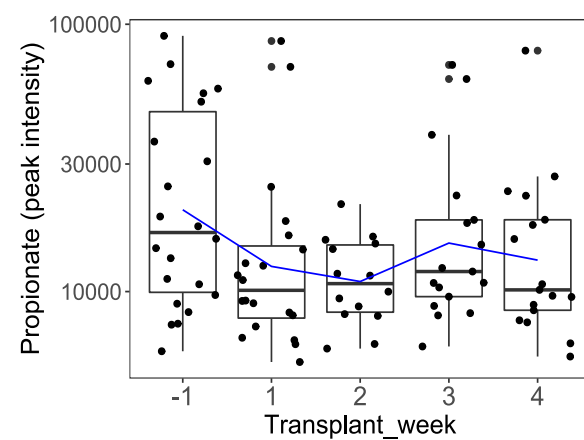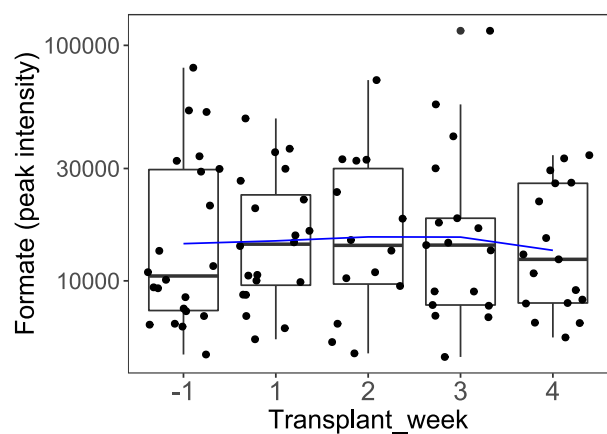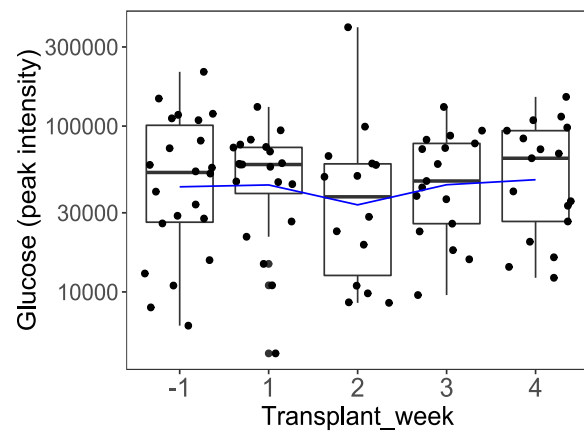

Supplement: Supplementary file 18 — Additional file 17: Figure S11. Metabolite profiles during the first five weeks of transplantation. Week -1 denotes days -7 to -1 relative to transplantation (sample range 19-26/week). The blue line indicates the mean for each week. [file 40168_2022_1270_MOESM18_ESM.pdf]

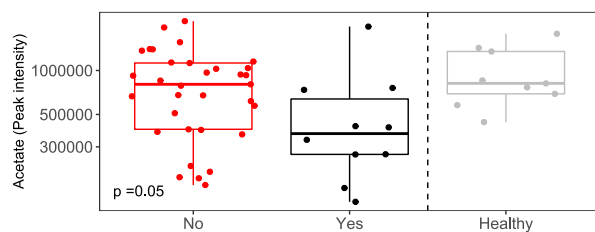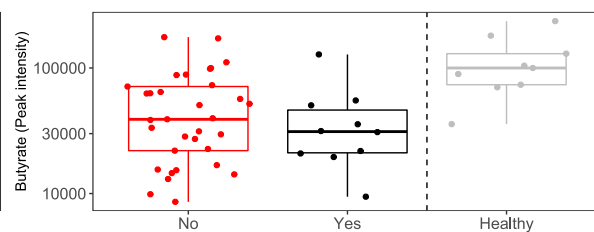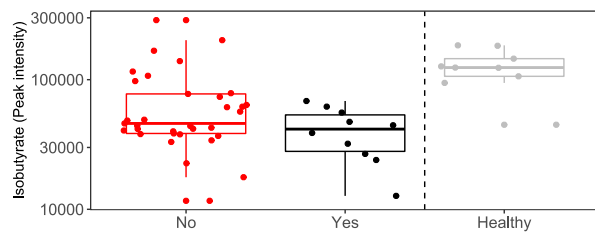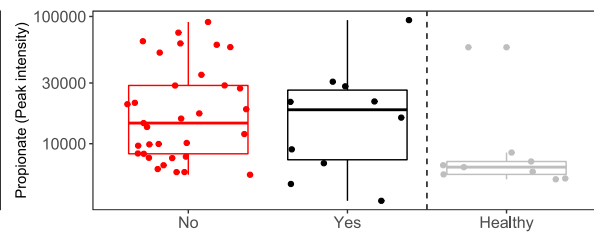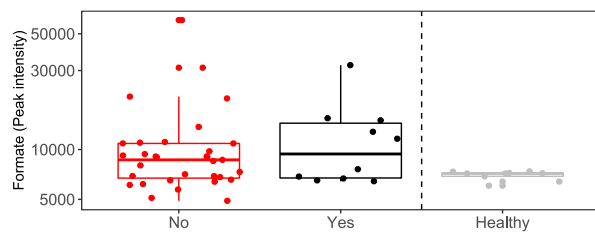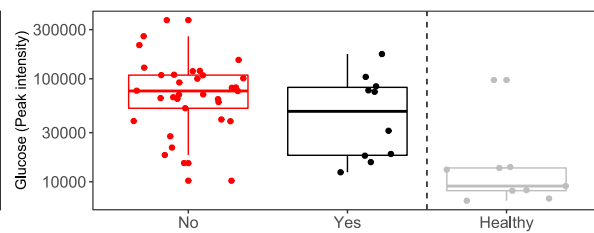

Supplement: Supplementary file 19 — Additional file 18: Figure S12. Metabolites at baseline in samples from allogeneic HSCT patients on (n = 33) and off (n = 10) total parenteral nutrition. Kruskal-Wallis univariate comparison. [file 40168_2022_1270_MOESM19_ESM.pdf]
