# Supplementary material for: Linking gastrointestinal microbiota and metabolome dynamics to clinical outcomes in paediatric haematopoietic stem cell transplantation
Source: Microbiome. 2022 Jun 10;10:89. doi: 10.1186/s40168-022-01270-7 (PMC9185888; doi:10.1186/s40168-022-01270-7)
Supplement: Supplementary file 10 — Additional file 9: Table S2. Univariate and multivariate Cox models with Enterococcus domination (>30%) as the dependent variable. The 95% CI and P values were estimated using the robust sandwich estimator. P value of <0.05 was considered significant. Abbreviations: CI, confidence interval; HR, hazard ratio; -, not significant. [file 40168_2022_1270_MOESM10_ESM.docx]

**Table S2 Univariate and multivariate Cox models with *Enterococcus* domination (>30%) as the dependent variable.** The 95% CI and P values were estimated using the robust sandwich estimator. P value of <0.05 was considered significant.

| **Univariate** | | | | **Multivariate** | | |
| --- | --- | --- | --- | --- | --- | --- |
| **Variables** | **HR** | **95% CI** | **p value** | **HR** | **95% CI** | **p value** |
| Age | 1 | 1-1.01 | 0.29 |  |  |  |
| Sex female: Yes | 0.61 | 0.4-0.93 | 0.32 |  |  |  |
| Diagnosis: Malignant | 0.63 | 0.43-0.94 | 0.19 |  |  |  |
| Conditioning: Myeloablative | 2.14 | 1.45-3.14 | 0.03 | 1.89 | 1.26-2.84 | 0.06 |
| More than one transplant: Yes | 1.71 | 1.02-2.86 | 0.13 |  |  |  |
| Cell source cord: Yes | 0.45 | 0.16-1.23 | 0.28 |  |  |  |
| Macrolides: Yes | 2.04 | 1.33-3.14 | 0.05 | 1.61 | 1.02-2.53 | 0.18 |
| Quinolones: Yes | 1.14 | 0.77-1.7 | 0.70 |  |  |  |
| BS beta lactams: Yes | 1.63 | 1.09-2.44 | 0.15 |  |  |  |

Abbreviations: CI, confidence interval; HR, hazard ratio; -, not significant.
