# Supplementary material for: Linking gastrointestinal microbiota and metabolome dynamics to clinical outcomes in paediatric haematopoietic stem cell transplantation
Source: Microbiome. 2022 Jun 10;10:89. doi: 10.1186/s40168-022-01270-7 (PMC9185888; doi:10.1186/s40168-022-01270-7)
Supplement: Supplementary file 12 — Additional file 11: Table S3. Univariate and multivariate Cox models with Viraemia as the dependent variable. The 95% CI and P values were estimated using the robust sandwich estimator. P value of <0.05 was considered significant. [file 40168_2022_1270_MOESM12_ESM.docx]

**Table S3 Univariate and multivariate Cox models with Viraemia as the dependent variable.** The 95% CI and P values were estimated using the robust sandwich estimator. P value of <0.05 was considered significant.

| Univariate | | | | Multivariate | | |
| --- | --- | --- | --- | --- | --- | --- |
| Variable | HR | 95% CI | p value | HR | 95% CI | p value |
| Sex female: Yes | 0.95 | 0.58-1.53 | 0.82 |  |  |  |
| Diagnosis: Malignant | 1.08 | 0.66-1.76 | 0.77 |  |  |  |
| Age | 0.74 | 0.35-1.56 | 0.42 |  |  |  |
| Serotherapy: Yes | 0.87 | 0.42-1.82 | 0.72 |  |  |  |
| Conditioning: Myeloablative | 1.19 | 0.7-2.03 | 0.51 |  |  |  |
| Cell source cord: Yes | 0.56 | 0.09-3.66 | 0.54 |  |  |  |
| More than one transplant: Yes | 1.59 | 1.01-2.52 | 0.05 | 1.44 | 0.93-2.23 | 0.1 |
| Microbiome CST: 2 | 1.32 | 0.73-2.36 | 0.36 | 1.28 | 0.71-2.32 | 0.41 |
| Microbiome CST: 3 | 2.19 | 1.27.3.76 | 0.01 | 2.07 | 0.29-2.52 | 0.01 |
| Shannon effective | 0.96 | 0.9.-1.03 | 0.27 |  |  |  |
