# Supplementary material for: Linking gastrointestinal microbiota and metabolome dynamics to clinical outcomes in paediatric haematopoietic stem cell transplantation
Source: Microbiome. 2022 Jun 10;10:89. doi: 10.1186/s40168-022-01270-7 (PMC9185888; doi:10.1186/s40168-022-01270-7)
Supplement: Supplementary file 14 — Additional file 13: Table S4. Univariate Cox model with Viraemia as the dependent variable and dominance as the independent variable. The 95% CI and P values were estimated using the robust sandwich estimator. P value of <0.05 was considered significant. Dominance is classified as >30% Relative abundance. [file 40168_2022_1270_MOESM14_ESM.docx]

**Table S4 Univariate Cox model with Viraemia as the dependent variable and dominance as the independent variable.** The 95% CI and P values were estimated using the robust sandwich estimator. P value of <0.05 was considered significant.

| **Variable** | **HR** | **95% CI** | **p value** |
| --- | --- | --- | --- |
| Enterococcus_dominant vs others | 1.45 | 0.92-2.29 | 0.11 |
| Enterobacteriaceae_ dominant vs others | 1.78 | 0.92-3.45 | 0.09 |
| Escherichia_Shigella_dominant vs others | 1.04 | 0.64-1.70 | 0.86 |
| Klebsiella_dominant vs others | 0.61 | 0.21-1.77 | 0.36 |
| Veillonella_dominant vs others | 1.23 | 0.77-1.96 | 0.40 |

| Dominance is classified as >30% Relative abundance |
| --- |
