# Supplementary material for: Linking gastrointestinal microbiota and metabolome dynamics to clinical outcomes in paediatric haematopoietic stem cell transplantation
Source: Microbiome. 2022 Jun 10;10:89. doi: 10.1186/s40168-022-01270-7 (PMC9185888; doi:10.1186/s40168-022-01270-7)
Supplement: Supplementary file 15 — Additional file 14: Table S5. Univariate Cox model with GvHD as the dependent variable. The 95% CI and P values were estimated using the robust sandwich estimator. P value of <0.05 was considered significant. [file 40168_2022_1270_MOESM15_ESM.docx]

**Table S5 Univariate Cox model with GvHD as the dependent variable.** The 95% CI and P values were estimated using the robust sandwich estimator. P value of <0.05 was considered significant.

| **Variable** | **HR** | **95% CI** | **P value** |
| --- | --- | --- | --- |
| Sex female: Yes | 0.90 | 0.47-1.72 | 0.75 |
| Diagnosis: Malignant | 0.70 | 0.37-1.13 | 0.26 |
| Age | 1.00 | 0.99-1.00 | 0.38 |
| Serotherapy: Yes | 0.82 | 0.28-2.42 | 0.71 |
| Graft manipulation: Yes | 0.69 | 0.16-3.09 | 0.63 |
| More than one transplant: Yes | 1.27 | 0.55-2.95 | 0.58 |
| Conditioning: Myeloablative | 1.21 | 0.66-2.21 | 0.53 |
| Shannon effective | 1.02 | 0.91-1.14 | 0.79 |
| Microbiome CST: 2 | 0.90 | 0.28-2.84 | 0.85 |
| Microbiome CST: 3 | 0.58 | 0.14-2.44 | 0.46 |
