# Supplementary material for: Linking gastrointestinal microbiota and metabolome dynamics to clinical outcomes in paediatric haematopoietic stem cell transplantation
Source: Microbiome. 2022 Jun 10;10:89. doi: 10.1186/s40168-022-01270-7 (PMC9185888; doi:10.1186/s40168-022-01270-7)
Supplement: Supplementary file 16 — Additional file 15: Table S6. A logistic regression model with viraemia as the dependent variable. P value of <0.05 was considered significant. [file 40168_2022_1270_MOESM16_ESM.docx]

**Table S6 A logistic regression model with viraemia as the dependent variable.** P value of <0.05 was considered significant.

| **Viraemia** | **Estimate (Standard error)** | **Pr(>\|z\|)** |
| --- | --- | --- |
| (Intercept) | -8.45E-05 (1.65) | 0.61 |
| Butyrate | -3.45E-05 (1.49E-05) | 0.02 |
| Glutamate | 1.21E-04 (6.55E-05) | 0.06 |
| Pyruvate | 5.54E-06 (3.989E-06) | 0.16 |
